# Simultaneously Engineering the Amorphous Phase and Branch Morphology of High‐Entropy Alloy Nanomaterials for Enhanced Ethylene Glycol Oxidation

**DOI:** 10.1002/adma.73384

**Published:** 2026-05-14

**Authors:** Biao Huang, Xinyu Chen, Jianbin Luo, Jing Cao, Deqi Fan, Min Bi, Fukai Feng, Lei Huang, Chin Yi En, Yu Zhang, Zhiheng Lyu, Ming Zhao

**Affiliations:** ^1^ Department of Materials Science and Engineering National University of Singapore Singapore Singapore; ^2^ School of Materials Science and Engineering University of Science and Technology Beijing Beijing China; ^3^ Centre for Hydrogen Innovations National University of Singapore Singapore Singapore; ^4^ School of Mechanical and Power Engineering East China University of Science and Technology Shanghai China; ^5^ Department of Chemical and Biological Engineering University of Alabama Tuscaloosa Alabama USA

**Keywords:** ethylene glycol oxidation, ethylene glycol oxidation‐assisted water electrolysis, high entropy alloy, morphology engineering, phase engineering

## Abstract

High entropy alloy (HEA) nanomaterials have emerged as an intriguing class of catalysts for diverse catalytic applications, yet engineering their morphology and/or crystal phase remains challenging. Herein, we successfully achieve the simultaneous engineering of both the amorphous phase and branch morphology of PdCuNiCoFe HEA nanomaterials. Excitingly, the synthetic protocol is effectively extended to synthesizing a library of amorphous Pd‐based nanobranches, including binary, ternary, and quaternary alloys. When employed as catalysts for ethylene glycol oxidation reaction (EGOR), the amorphous PdCuNiCoFe HEA achieves superior activity and selectivity of glycolic acid approaching 98.6%, and enables a remarkable overpotential drop of 774 mV at 100 mA cm^‒2^ in EGOR‐assisted water electrolysis. Mechanistic investigation demonstrates the excellence of amorphous PdCuNiCoFe HEA in suppressing C−C cleavage, facilitating ^*^OH/^*^EG adsorption, and lowering the free energy requirement for glycolic acid production. This study offers a promising means to rationally engineer the morphology and phase of HEAs for enhanced electrocatalysis.

## Graphical abstract

Amorphous PdCuNiCoFe high‐entropy alloy nanobranches were developed through a versatile wet‐chemical synthetic strategy enabling simultaneous control of composition, phase, and morphology. The resulting amorphous PdCuNiCoFe nanobranches exhibit outstanding electrocatalytic performances for ethylene glycol oxidation (EGOR), delivering high selectivity toward glycolic acid (GA) and significantly reduced overpotential for EGOR‐assisted water electrolysis.

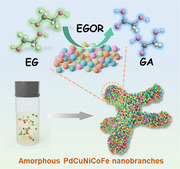

## 1 Introduction

High‐entropy alloy (HEA) nanomaterials, characterized by their high‐entropy effect, lattice distortion effect, sluggish diffusion effect, and cocktail effect [1], have recently shown great promise in electrocatalysis [2, 3, 4, 5], owing to their capabilities in lowering reaction energy barriers, optimizing intermediate adsorption, facilitating reaction kinetics, and reinforcing structural stability [6, 7, 8, 9, 10]. For example, Hu and co‐workers reported the carbothermal shock synthesis of PtPdRhRuCe HEA nanoparticles as catalysts for ammonia oxidation, exhibiting greatly enhanced selectivity for NO_x_ as compared to phase‐separated PtPdRhRuCe nanoparticles [11]. Wu et al. developed the synthesis of PtRuFeCoNi HEA‐based core‐shell nanocrystals for highly efficient hydrogen evolution and oxidation reactions, wherein the cocktail effect in HEA atomic layers was confirmed to play a vital role in optimizing the hydrogen adsorption during catalysis [12]. Despite these merits, a majority of HEA nanomaterials synthesized using conventional high‐temperature/shock‐cooling methods typically feature a spheroidal Wulff shape with a minimized surface energy and a certain crystal phase at elevated temperatures owing to the thermodynamic constraint [11, 13, 14]. This dilemma retards the structural engineering of HEA catalysts for further performance optimization and thus impedes their implementation into broad electrocatalytic reactions.

One effective strategy to address the above challenge relies on the phase engineering of nanomaterials (PEN) [15, 16, 17, 18], wherein the atom arrangement is spatially regulated to tune the electronic structure and catalyst‐adsorbate interaction for enhanced electrocatalysis [19, 20, 21, 22]. As a compelling example, Zhang et al. reported the preparation of unconventional hexagonal close‐packed PdCu nanoparticles, achieving notably improved oxygen reduction reaction activity than the conventional face‐centered cubic (fcc) counterpart. In particular, an amorphous phase with disordered atomic arrangements can expose unsaturated coordination sites that are highly active for catalysis [23, 24]. Another promising strategy is to engineer the morphology of HEA nanomaterials, given that the distinct surfaces exposed by different morphologies essentially define their interaction with reaction species and thus determine the catalytic performance [4, 25, 26, 27]. As a compelling example, Guo and co‐workers demonstrated the synthesis of PdPtNiCuZn HEA nanosheets, in which the tensile strain intrinsic to the nanosheet structure significantly enhanced the activity for the methanol oxidation reaction, surpassing conventional nanoparticle structure [28]. Branched morphologies have also been shown to increase the accessible surface area, expose more active sites, facilitate mass transport, and enhance structural stability, leading to improved electrocatalytic performances in electrocatalysis [29, 30, 31]. Nonetheless, it remains challenging to obtain an HEA structure while maintaining a tight control over the morphology and/or crystal phase due to the intrinsically heterogeneous crystallization behaviors of various elements, which hinders the exploration of structure‐performance relations and the development of high‐performance HEA catalysts.

Here, we develop a novel wet‐chemical synthesis to simultaneously engineer both the amorphous crystal phase and branch morphology of PdCuNiCoFe HEA nanomaterials. The morphology evolution and formation mechanism of HEA nanobranches were clarified, wherein Pd and Cu play key roles in defining the branch morphology and amorphous phase. Moreover, our synthetic protocol could be readily extended to produce a series of amorphous Pd‐based alloy nanobranches, including binary, ternary, and quaternary alloys, offering a promising platform for investigating the structure‐performance relationship in HEA catalysis. Both the crystal and electronic structures of the PdCuNiCoFe HEA nanobranches were explicitly examined. Excitingly, when employed as catalysts for the selective electrochemical ethylene glycol oxidation reaction (EGOR) to glycolic acid (GA) in alkaline electrolytes, the PdCuNiCoFe HEA catalyst outperforms other alloy counterparts and commercial Pd/C in activity and stability as well as GA selectivity. When coupling EGOR with water electrolysis, the overpotentials to achieve various current densities for green hydrogen production were notably reduced, in both a two‐electrode reactor and an anion exchange membrane water electrolyzer (AEMWE), with a durable long‐term operation for 300 h. In situ spectroscopic analyses and density functional theory (DFT) calculations were also conducted to investigate the reaction mechanisms of EGOR on various catalysts.

## 2 Results and Discussion

### 2.1 Synthesis and Characterization of Amorphous HEA Nanobranches

**FIGURE 1:**
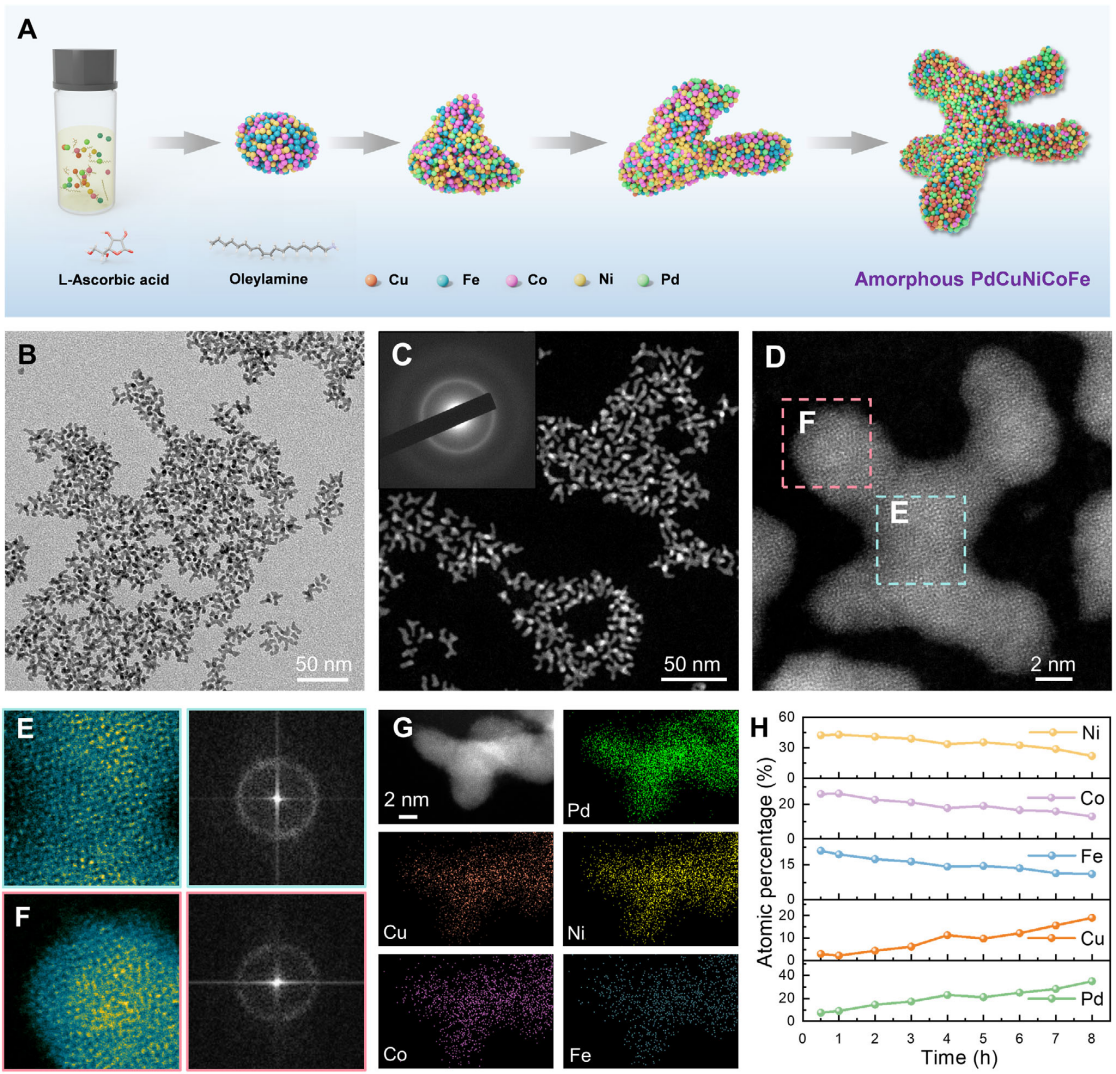
Synthesis and characterization of HEA nanobranches. (A) Schematic illustrating the synthesis of amorphous PdCuNiCoFe HEA nanobranches. (B) TEM and (C) STEM images of amorphous PdCuNiCoFe HEA nanobranches. The inset in C shows the SAED pattern. (D) HAADF‐STEM image of a representative amorphous PdCuNiCoFe HEA nanobranch. (E and F) Enlarged HAADF‐STEM image with the corresponding FFT pattern of the areas marked in (D). (G) STEM image and EDS elemental mapping results of a representative amorphous PdCuNiCoFe HEA nanobranch. (H) Atomic percentages of various elements in the intermediate products collected at different reaction periods of the standard synthesis.

As schematically illustrated in Figure 1A (see Methods in the Supporting Information for details), the amorphous PdCuNiCoFe HEA nanobranches were synthesized via a one‐pot, wet‐chemical reduction of various metal precursors in the presence of oleylamine (OAm) as the solvent and L‐Ascorbic acid (AA) as the reductant. The transmission electron microscopy (TEM) and scanning TEM (STEM) images of the PdCuNiCoFe product display a branch morphology and a uniform distribution (Figure 1B,C, as well as Figures S1 and S2), with an average branch length of 8 ± 1 nm (Figure S3). Notably, the nanobranches were found to possess an amorphous phase, according to the diffuse rings in the selected area electron diffraction (SAED) pattern (Figure 1C inset). The amorphous phase was further confirmed by the high‐angle annular dark‐field STEM (HAADF‐STEM) analysis (Figure 1D), where various metal atoms are packed in a disordered manner. The diffuse rings in the corresponding fast Fourier transform (FFT) patterns of the center (Figure 1E) and branch (Figure 1F) domains of a representative PdCuNiCoFe nanobranch validate the amorphous phase across the whole particle. Moreover, although the number of branches in the products varies slightly from particle to particle, all the PdCuNiCoFe nanobranches share the same amorphous phase (Figure S4). The energy dispersive X‐ray spectroscopy (EDS) elemental mapping analysis of the PdCuNiCoFe nanobranches indicates the homogeneous distributions of Pd, Cu, Ni, Co, and Fe atoms (Figure 1G; Figure S5), suggesting the formation of an alloy structure. Especially, the atomic percentages of Pd, Cu, Ni, Co, and Fe were determined as 35%, 19%, 22%, 13%, and 11%, respectively, validating an HEA structure (Table S1).

To understand the formation mechanism of the amorphous PdCuNiCoFe HEA nanobranches, we first examined the time‐dependent morphological evolution of the intermediates using TEM (Figure S6). In the initial stage of synthesis, i.e., within 2 h, small nanoparticles with growing sizes and an amorphous phase were generated (Figure S6A,B). As the synthesis was prolonged to 3 h, branches started to grow on the nanoparticles while their length and number were gradually enlarged (Figure S6C–G). Eventually, uniform products with multi‐branches were obtained (Figure S6H). Noticeably, the amorphous phase was well maintained for all intermediate products throughout the morphological evolution, as demonstrated by the corresponding SAED patterns (Figure S6).

**FIGURE 2:**
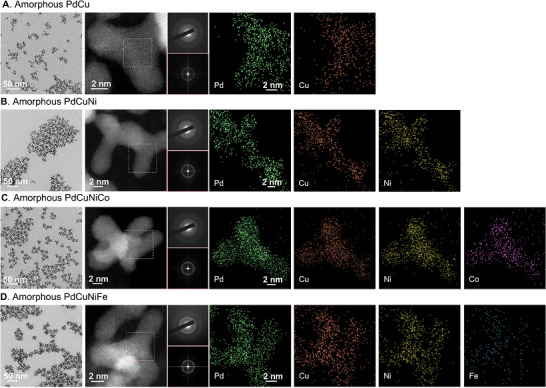
Synthesis and characterizations of a library of amorphous nanobranches. (A–D) TEM, HAADF‐STEM, SAED, FFT, and EDS mapping results of amorphous PdCu (A), PdCuNi (B), PdCuNiCo (C), and PdCuNiFe (D) nanobranches.

In addition, the atomic percentages of various elements during the reaction process were tested by inductively coupled plasma optical emission spectroscopy (ICP‐OES), as presented in Figure 1H. After 1 h of reaction, the combined atomic percentage of Ni (43%), Co (26%), and Fe (19%) reached approximately 90%, indicating the dominant reduction of Ni, Co, and Fe precursors at the beginning of the synthesis to form amorphous alloy nanoparticles. As the reaction proceeded, the atomic percentages of Ni, Co, and Fe decreased gradually while those of Pd and Cu progressively increased to 35% and 19%, respectively, in the final amorphous PdCuNiCoFe HEA product. To understand the explicit role of each metal precursor in the formation of a branch morphology and an amorphous phase, we conducted a series of control experiments. When only one type of metal precursor was used for the synthesis, no products were obtained (Figure S7). However, a variety of products were formed when mixing the Pd precursor with another type of metal precursor (Figure 2A; Figure S8). In particular, the PdCu nanoparticles also indicate a branch morphology and an amorphous phase, whereas PdNi, PdCo, and PdFe nanoparticles show a non‐branched morphology, with SAED patterns confirming their crystalline fcc phase (Figure S8), validating the critical role of PdCu in defining both the morphology and crystal phase. Moreover, the removal of Ni, Co, or Fe precursors from the standard synthesis does not affect the branch morphology, whereas the absence of Cu precursor yielded irregular nanoparticles (Figure S9), further confirming the morphological origin of nanobranches from PdCu. It is worth pointing out that although Pd and Cu significantly influence the formation of amorphous nanobranches, their atomic percentages were <10% within 1 h of synthesis. We attributed this phenomenon to the accelerated reduction kinetics of other metal precursors catalyzed by the initially formed Pd owing to the highest reduction potential of Pd^2+^ (Pd^2+^/Pd = 0.987 V vs. standard hydrogen electrode) [32], as suggested by the sharp contrast of reducing monometallic and Pd‐based bimetallic precursors (Figure 2A and Figure S8). Moreover, upon the generation of Fe, Co, and Ni atoms, galvanic replacement of these atoms with Pd and Cu ions could also be thermodynamically favorable, which promotes the incorporation of Pd and Cu atoms to enable their homogeneous distributions in the final products.

### 2.2 Synthesis of a Library of Amorphous Nanobranches

Leveraging the functionality of PdCu in directing the morphology and atom packing, we further synthesized a library of PdCu‐based amorphous nanobranches ranging from binary to quaternary elements, including PdCu, PdCuNi, PdCuCo, PdCuFe, PdCuNiCo, PdCuNiFe, and PdCuCoFe (Figure 2; Figure S10). As shown in Figure 2, TEM images indicate the well‐defined branch morphology of all products, identical to that of PdCuNiCoFe HEA. The homogenous distribution of each element in these nanobranches indicates the successful construction of an alloyed structure. The amorphous crystal phase is also verified by the random arrangement of metal atoms in the HAADF‐STEM images and the diffused rings in the corresponding SAED and FFT patterns. The above results suggest the generality of the developed approach to synthesizing a library of amorphous Pd‐based alloy nanobranches ranging from binary to HEA.

### 2.3 Crystal and Electronic Structures Examination

**FIGURE 3:**
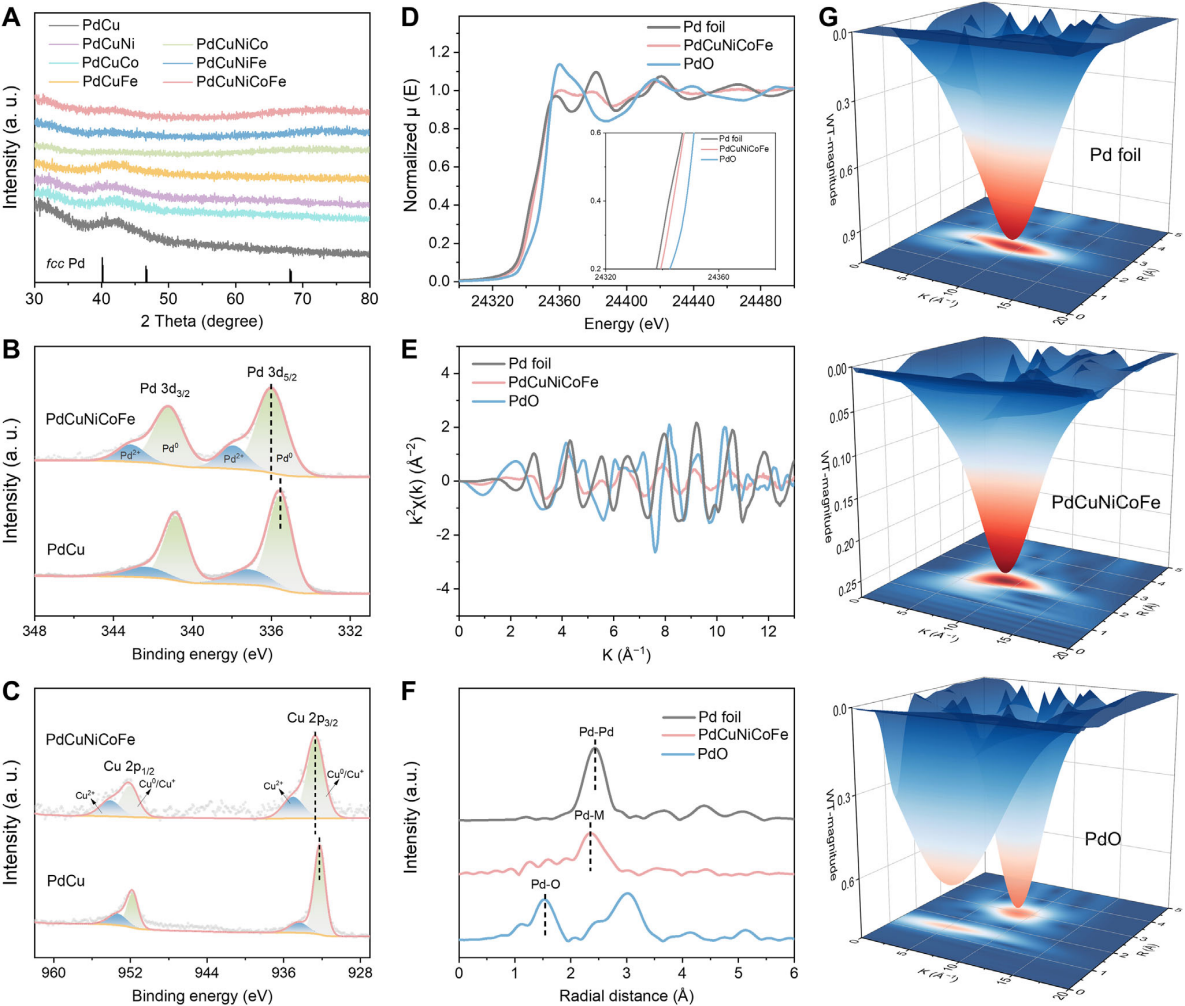
Crystal and electronic structures of amorphous HEA nanobranches. (A) XRD patterns of amorphous nanobranches with various compositions. The black lines mark the characteristic peak positions of crystalline Pd with an fcc phase. (B) Pd 3d and (C) Cu 2p XPS spectra of amorphous PdCuNiCoFe HEA and PdCu. (D) Pd *K*‐edge XANES spectra, (E) EXAFS spectra, (F) FT‐EXAFS spectra, and 3D WT‐EXAFS (G) of amorphous PdCuNiCoFe HEA, Pd foil, and PdO.

To examine the crystal phase at the bulk level, we conducted X‐ray diffraction (XRD) analysis on samples with various compositions, all of which display an amorphous phase (Figure 3A), evident by the absence of characteristic diffraction peaks for crystals. The valence states of the as‐obtained amorphous Pd‐based alloys were studied using X‐ray photoelectron spectroscopy (XPS). The Pd 3d XPS spectra of amorphous PdCuNiCoFe HEA and amorphous PdCu (Figure 3B), as well as other Pd‐based alloys (Figures S11–S14), indicate that Pd predominantly exists in the metallic state with the co‐existence of a small portion of oxidized Pd^2+^. Notably, the Pd^0^ peaks at 336.0 and 341.2 eV observed in the amorphous PdCuNiCoFe are positively shifted as compared to those of amorphous PdCu (335.6 and 340.8 eV) and pure Pd^0^ reported in the literature (335.2 and 340.5 eV) [33], indicating the electron transfer from Pd to neighbouring atoms in the amorphous PdCuNiCoFe HEA and thus potentially benefiting the catalyzing of oxidation reactions. A similar phenomenon was also observed in the Cu 2p XPS spectra (Figure 3C; Figures S12–S14), where Cu^0^/Cu^+^ dominates the Cu composition while a small portion of Cu^2+^ species appear, together with the positive shift of Cu^0^/Cu^+^ peaks in the HEA relative to the PdCu counterpart (Figure 3C). Moreover, the high‐resolution Ni 2p, Co 2p, and Fe 2p XPS spectra of amorphous PdCuNiCoFe HEA (Figure S11), amorphous PdCuNiFe (Figure S12), PdCuNiCo (Figure S13), and PdCuNi (Figure S14) all suggest the contribution from both metallic and oxidized states of Pd, Ni, Co, and Fe elements. We attribute the existence of oxidized states to the vulnerable oxidation of Cu, Ni, Co, and Fe upon exposure to air during sample washing and preparation.

To further investigate the electronic configuration, synchrotron X‐ray absorption spectroscopy (XAS) was carried out on amorphous PdCuNiCoFe HEA. The Pd *K*‐edge X‐ray absorption near‐edge structure (XANES) results (Figure 3D) demonstrate that the absorption edge for Pd in PdCuNiCoFe HEA is close to that of Pd foil and far away from that of PdO reference samples, suggesting the dominant metallic Pd in PdCuNiCoFe HEA and consistent with the XPS results. Moreover, the near‐edge absorption energies of Pd in PdCuNiCoFe HEA show a slight shift toward a higher energy relative to the Pd foil, indicating a higher valence state of Pd due to electron redistribution between Pd and neighboring atoms. Also, the Pd *K*‐edge extended X‐ray absorption fine structure (EXAFS) spectra of PdCuNiCoFe HEA are distinct from those of Pd foil and PdO references (Figure 3E), which suggests distinctive electronic configurations stemming from the formation of a well‐defined HEA structure [27]. In addition, the Fourier transform (FT)‐EXAFS (Figure 3F) and wavelet‐transformed 3D (WT)‐EXAFS (Figure 3G) analyses of PdCuNiCoFe HEA show a prominent peak at around 2.34 Å that corresponds to the Pd─M bond (M represents Pd, Cu, Ni, Co, or Fe; Figure 3F,G). The lower value of Pd‐M relative to Pd‐Pd (2.44 Å in Pd foil) was due to the disordered atomic packing in the amorphous PdCuNiCoFe HEA [34]. The XANES spectra of Cu, Ni, Co, and Fe demonstrate the coexistence of both metallic and oxidized states in these elements (Figure S15), which is likely due to the susceptible oxidation of surface Cu/Ni/Co/Fe when exposed to air [12, 35, 36].

### 2.4 Electrochemical Measurements

**FIGURE 4:**
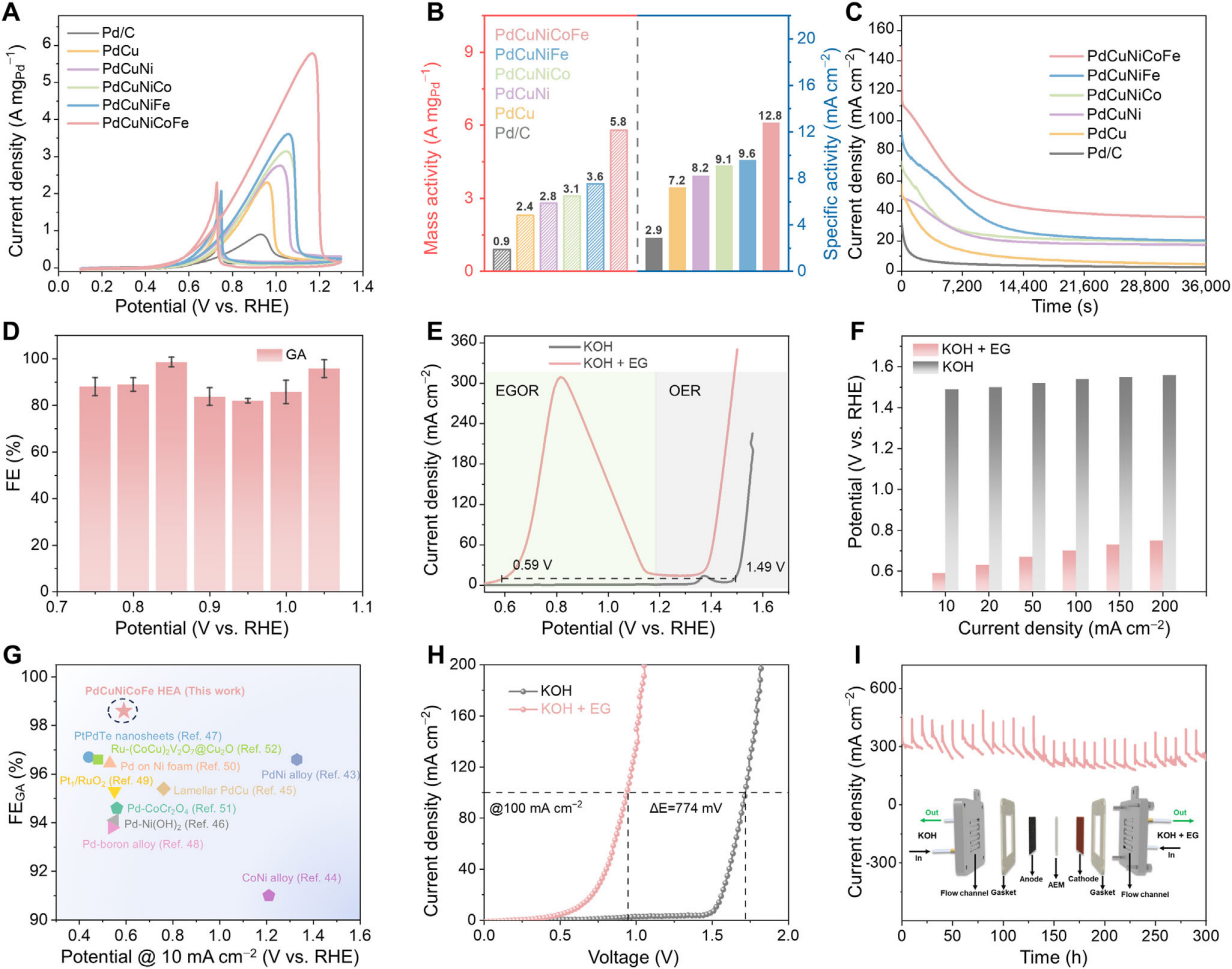
Electrochemical measurements. (A) CV curves of various catalysts recorded in N_2_‐saturated aqueous electrolytes containing 1.0 M KOH and 1.0 M EG. (B) Mass and specific activities of various catalysts. (C) Chronoamperometry curves at 0.82 V of various catalysts. (D) FEs of GA over PdCuNiCoFe HEA under different potentials. (E) LSV curves over PdCuNiCoFe HEA in N_2_‐saturated 1 M KOH with and without 1 M EG. Note that the small peak at around 1.38 V originates from the oxidation of the transition metal. (F) Comparisons of potentials required to reach different current densities for EGOR and OER over PdCuNiCoFe HEA. (G) Comparison of onset potential and FE of GA over the PdCuNiCoFe HEA catalyst with reported EGOR electrocatalysts in alkaline solutions. (H) LSV curves over PdCuNiCoFe HEA || Pt/C two‐electrode system in 1 M KOH with and without 1 M EG. (I) Chronoamperometry curves of the PdCuNiCoFe HEA || Pt/C MEA‐based AEMWE device at around 1.7 V. The electrolyte was refreshed every 10 h. The inset shows a schematic diagram of the AEMWE device.

The selective electrooxidation of EG, a major downstream product of poly(ethylene terephthalate) (PET) recycling, into GA has emerged as an attractive pathway for upcycling plastic waste under mild conditions [37]. Especially, when EGOR is coupled with water electrolysis, it allows the synchronous production of green hydrogen at improved energy efficiency due to the remarkably lower overpotential of EGOR relative to oxygen evolution reaction (OER) [38]. As Pd‐based catalysts are well reported to show outstanding performance toward EGOR [39], we then employed various types of amorphous Pd‐based alloys as catalysts for EGOR and benchmarked against the commercial Pd/C (Figure S16). Figure 4A shows the cyclic voltammetry (CV) curves of the catalysts obtained in N_2_‐saturated 1.0 M potassium hydroxide (KOH) and 1.0 M EG mixed solution. Specifically, the PdCuNiCoFe HEA nanobranches display a Pd mass‐normalized current density of 5.8 A mg_Pd_^−1^, which is 1.6, 1.9, 2.1, 2.4, and 6.4 times as high as that of PdCuNiFe (3.6 A mg_Pd_^−1^), PdCuNiCo (3.1 A mg_Pd_^−1^), PdCuNi (2.8 A mg_Pd_^−1^), PdCu (2.4 A mg_Pd_^−1^), and Pd/C catalysts (0.9 A mg_Pd_^−1^), respectively (Figure 4B). Then, we evaluated the electrochemically active surface areas (ECSAs) through examining the PdO peak areas in the CV curves tested in N_2_‐saturated 1.0 M KOH (Figure S17) [40]. The derived ECSAs show that the diverse types of nanobranches all display higher ECSAs than that of the commercial Pd/C, with the HEA nanobranches having the highest ECSA (Figure S18A). The ECSA‐normalized activity, i.e., specific activity, of the HEA nanobranches (12.8 mA cm^−2^) is 4.4 times greater than that of Pd/C (2.9 mA cm^−2^, Figure 4B; Figure S18B), lower than the 6.4‐fold enhancement in mass activity. Considering the approximate 1.5‐fold enhancement in ECSA of HEA relative to Pd/C, the superb mass activity shows a larger enhancement than the specific activity, indicating that the improved performance is dominantly contributed by the enhanced intrinsic activity of active sites, with a secondary contribution from the increase in the number of active sites.

To probe the adsorption behavior of EG [41], open‐circuit potential (OCP) measurements were conducted on different catalysts (Figure S19A). Upon introducing 1 M EG into the 1 M KOH electrolyte, the HEA catalyst exhibits a more significant potential drop of 0.58 V than the amorphous PdCu catalyst (0.5 V) and commercial Pd/C (0.47 V), indicating stronger EG adsorption on the HEA catalyst. Moreover, electron paramagnetic resonance (EPR) measurements were performed to probe ^*^OH adsorbates and possible reaction intermediates, using 5,5‐dimethyl‐1‐pyrroline N‐oxide (DMPO) as a spin‐trapping agent. In a pure KOH electrolyte, the HEA catalyst displays the most intense quartet signals corresponding to DMPO‐•OH adducts (Figure S19B), suggesting enhanced interaction between the catalyst surface and OH^−^ species, which promotes the oxidation of OH^−^ to form •OH radicals. Chronoamperometric measurements at 0.82 V vs. reversible hydrogen electrode (RHE) also demonstrate the superior stability of the HEA nanobranches, which maintains 66.1% and 23.9% of the initial current density after 1,800 and 36,000 s, respectively, remarkably higher than 28.4% and 8.1% of the commercial Pd/C (Figure 4C). The superior catalytic stability of the PdCuNiCoFe HEA catalyst could be rationalized by the structural stability, where the branch morphology, amorphous phase, and elemental composition after the stability test all remain intact (Figure S20 and Table S1). When benchmarked against the reported catalysts, the PdCuNiCoFe HEA nanobranches represent one of the best Pd‐based electrocatalysts for EGOR in alkaline electrolyte (Table S2). The superb electrochemical activity and stability of the HEA catalyst could be attributed to the synergistic effects of its branched morphology, amorphous phases, and high entropy composition. The branched architecture provides a high surface area and abundant edge/channels, thus facilitating better active site exposure and reactant accessibility [30]. Amorphous phases with long‐range disordered structures introduce abundant unsaturated coordination sites for reactions [23], while the high entropy composition with sluggish diffusion effect stabilizes the catalyst against dissolution and phase segregation [42].

The selective conversion of EG into GA is highly desired owing to the superior economic value of GA. To this end, we also examined the product selectivity of the PdCuNiCoFe HEA nanobranches toward EGOR at 0.75–1.05 V vs. RHE, where the current density is considerable. The ^1^H nuclear magnetic resonance (NMR) results of the electrolytes before and after the chronoamperometric measurements suggest that GA is the major liquid product during EGOR (Figure S21), whose concentration could be determined by referring to a pre‐measured standard curve (Figure S22). Significantly, the amorphous HEA nanobranches exhibit Faradaic efficiencies (FEs) of >80% at various potentials for selective GA production (Figure 4D), and could reach 98.6% at 0.85 V vs. RHE, showing great promise for practical applications and is among the highest values reported for alkaline EGOR recently (Table S2). The yield rates of GA were also calculated (Figure S23), where HEA nanobranches show a high GA yield rate of 1.52 mmol cm^−2^ h^−1^ at 1.05 V vs. RHE.

Given the remarkably lower thermodynamic potential of EGOR (0.57 V vs. RHE) than OER (1.23 V vs. RHE), we also substituted EGOR for OER as the anodic reaction for green H_2_ production via water electrolysis. As shown in Figure 4E, linear sweep voltammetry (LSV) tests suggest that in the presence of anodic EGOR, the amorphous HEA nanobranches display a low onset potential of 0.59 V vs. RHE at 10 mA cm^−2^, with a substantially reduced overpotential of 900 mV at 10 mA cm^−2^ relative to OER (0.59 vs. 1.49 V) toward water electrolysis. Moreover, in the current density range from 10 to 200 mA cm^−2^, the overpotentials of EGOR all show >700 mV decay than those of OER (Figure 4F), validating the strength of EGOR in improving the energy efficiency of water electrolysis for green H_2_ production. Notably, PdCuNiCoFe HEA displays superior activity compared with the Pd‐based non‐HEA catalysts (Figure S24), delivering a 4.3‐ and 7.5‐fold higher current density relative to PdCu and commercial Pd/C catalyst, respectively. The low onset potential of 0.59 V and high FE of GA (98.6%) over the PdCuNiCoFe HEA catalyst are also among the best reported state‐of‐the‐art EGOR catalysts (Figure 4G) [43, 44, 45, 46, 47, 48, 49, 50, 51, 52].

To assess the potential of EGOR‐assisted water electrolysis for practical applications, we first fabricated a membrane‐free two‐electrode reactor, employing amorphous PdCuNiCoFe HEA nanobranches as the anode and commercial Pt/C as the cathode (denoted as PdCuNiCoFe HEA || Pt/C). The LSV curves (Figure 4H) reveal that when coupling EGOR with HER, PdCuNiCoFe HEA || Pt/C requires a potential reduced by 774 mV than that needed for conventional overall water electrolysis to achieve 100 mA cm^−2^. To further advance the practical applicability of the EGOR‐assisted water electrolysis system, an AEMWE device equipped with a membrane‐electrode‐assembly (MEA) was constructed (Figure S25). Apparently, the as‐assembled PdCuNiCoFe HEA || Pt/C AEMWE device shows higher current densities in the EGOR+HER system as compared to that in the conventional OER+HER system (Figure S26), showing a cell voltage 300 mV lower than that in the OER+HER system at 500 mA cm^−2^ (1.66 V vs. 1.96 V). The stability of the PdCuNiCoFe HEA || Pt/C AEMWE device was evaluated by repeating chronoamperometric measurements at around 1.7 V every 10 h (Figure 4I). In each 10‐h cycle of test, the current density shows a sharp decrease in the initial stage, followed by a slow decay due to the rapid consumption of EG and poisoning of ^*^CO intermediates on the anode surface. Notably, the activity of PdCuNiCoFe HEA || Pt/C AEMWE could be restored by refreshing the electrolyte after each cycle. During the long‐term durability test, PdCuNiCoFe HEA || Pt/C AEMWE exhibits consistently high current density of 200–300 mA cm^−2^ with insignificant decline for 300 h, validating its outstanding stability in practical applications.

### 2.5 Catalytic Mechanism Investigation

**FIGURE 5:**
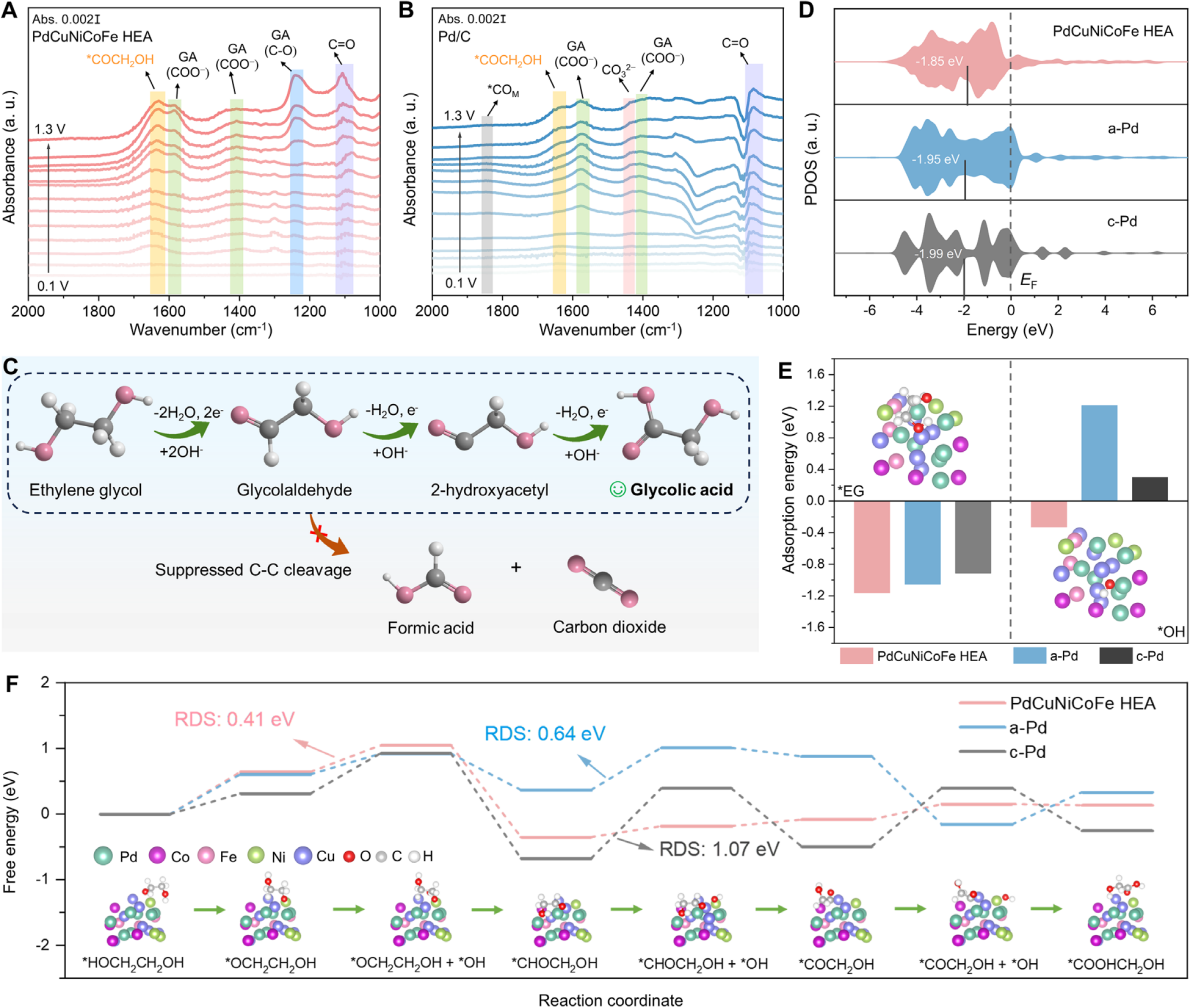
In situ characterizations and DFT calculations. (A,B) In situ ATR‐SEIRAS recorded at different potentials of EGOR catalyzed by the PdCuNiCoFe HEA (A) and commercial Pd/C (B). (C) Proposed EGOR pathway over the PdCuNiCoFe HEA catalyst. (D) The PDOSs of the PdCuNiCoFe HEA, amorphous Pd (a‐Pd), and crystalline Pd (c‐Pd) surfaces. The solid vertical lines mark the positions of the *d*‐band center. (E) Adsorption energy of ^*^EG and ^*^OH over the PdCuNiCoFe HEA, a‐Pd, and c‐Pd surfaces. Inset is the surface adsorption model of ^*^EG and ^*^OH over the PdCuNiCoFe HEA. (F) Free energy diagrams for EGOR over the PdCuNiCoFe HEA, a‐Pd, and c‐Pd surfaces.

To rationalize the reaction mechanism, in situ attenuated total reflection surface‐enhanced infrared absorption spectroscopy (ATR‐SEIRAS) analysis under operando conditions on various catalysts was conducted. Specifically, the peak at ∼1093 cm^−1^ associated with C═O stretch was observed on various catalysts (Figure 5A,B, as well as Figures S27–S30), suggesting the initial oxidation of EG molecules into glycolaldehyde [53, 54]. At 0.3 V, the characteristic peak of 2‐hydroxyacetyl (^*^COCH_2_OH) was observed on the PdCuNiCoFe HEA at 1631 cm^‒1^, which stems from the deprotonation of glycolaldehyde and is a key intermediate that could subsequently couple with ^*^OH to form GA. Distinctively, the same peak was observed at a higher potential for the Pd/C catalyst (0.6 V) and other Pd‐based alloy catalysts (0.5–1.1 V; Figures S27–S30), confirming that the amorphous HEA nanobranches could effectively promote the generation of ^*^COCH_2_OH species and accelerate the catalytic kinetics. Notably, the characteristic bands associated with the C−O stretching (1240 cm^−1^), and with the symmetric (1405 cm^−1^) and antisymmetric stretching (1584 cm^−1^) of COO^−^ in glycolate were well resolved [55, 56], indicating the ultimate formation of GA upon further oxidation of 2‐hydroxyacetyl. In situ Raman spectra also confirm the formation of GA on PdCuNiCoFe HEA (Figure S31), as indicated by the characteristic peaks at 1065 and 1465 cm^−1^ that correspond to COO^−^ of GA, respectively. It is worth emphasizing that on PdCuNiCoFe HEA nanobranches, we did not observe the characteristic peaks of species related to the formation/dissolution of CO_2_, such as the multiple‐bonded CO (1840 cm^−1^) formed by C−C cleavage and CO_3_^2−^ (1435 cm^−1^) generated from CO_2_ dissolution. However, these peaks were clearly detected on the commercial Pd/C (Figure 5B). This sharp contrast suggests that the C−C cleavage was largely suppressed on the PdCuNiCoFe HEA catalyst during EGOR, which is key to promoting the selective GA production. Building on these results, we propose the oxidation pathway of EG on the amorphous HEA nanobranches (Figure 5C) as follows [52, 53]:

$$\begin{array}{c} {}*{\mathrm{HOCH}}_{2}{\mathrm{CH}}_{2}\mathrm{OH}\to *{\mathrm{OCH}}_{2}{\mathrm{CH}}_{2}\mathrm{OH}\overset{*\mathrm{OH}}{\to }*{\mathrm{CHOCH}}_{2}\mathrm{OH}\overset{*\mathrm{OH}}{\to }\\ {}*{\mathrm{COCH}}_{2}\mathrm{OH}\overset{*\mathrm{OH}}{\to }*{\mathrm{COOHCH}}_{2}\mathrm{OH}\left(\mathrm{GA}\right) \end{array}$$

DFT calculations were further conducted to unravel the origin of the excellent catalytic performances of PdCuNiCoFe HEA nanobranches, in which three structural surfaces (Figures S32 and S33) were constructed, including PdCuNiCoFe HEA, amorphous Pd (denoted as a‐Pd), and crystalline Pd (denoted as c‐Pd). The projected density of states (PDOS) analysis shows that the *d*‐band center of Pd (Figure 5D) shifts 0.04 eV closer to the Fermi level when the crystal phase is switched from crystalline to amorphous, while an additional 0.10 eV shift is further observed for the HEA nanobranches, suggesting the dominant role of an HEA structure in regulating the electronic structure for promoting reaction species adsorption during EGOR. This statement was confirmed by evaluating the adsorption of ^*^EG and ^*^OH, two key species associated with the initial EG oxidation and dehydrogenation of intermediates for GA formation, respectively, on various catalyst surfaces. Compared with c‐Pd and a‐Pd surfaces, PdCuNiCoFe HEA shows the strongest adsorption affinity toward both ^*^EG and ^*^OH (Figure 5E), which is beneficial to the initial EG oxidation and eventual GA formation via dehydrogenation of ^*^COCH_2_OH, respectively. The *d*‐band center is employed here as a qualitative descriptor to provide insight into the adsorption behavior, rather than as a strict quantitative predictor. Furthermore, a linear scaling plot was introduced to further analyze the relationship between the *d*‐band center and the adsorption strength (Figure S34). The results show that the adsorption strength of EG increases as the *d*‐band center moves closer to the Fermi level, which confirms the trend observed in our analysis: stronger adsorption correlates with the upshift of *d*‐band center. Despite the enhanced adsorption strength on PdCuNiCoFe HEA, the superior catalytic activity of PdCuNiCoFe HEA during EGOR suggests that the HEA surface is located near the optimal region of the activity volcano, rather than in the over‐binding regime, which could be benefited from the high active site density of the amorphous and high entropy structure. Furthermore, the charge density difference and Bader charge analyses were conducted for three surfaces with adsorbed ^*^EG (Figure S35). Notably, the PdCuNiCoFe HEA displays the largest charge transfer (0.49 e^‒^) between the Pd active site and the ^*^EG reactant, exceeding those observed for a‐Pd (0.37 e^‒^) and c‐Pd (0.25 e^‒^), validating that the introduction of amorphous phase and high entropy composition collectively promotes interfacial electron exchange with ^*^EG, thereby accelerating the EGOR kinetics. Moreover, according to the detailed EGOR to GA pathway discussed above, the adsorption surfaces of key intermediates on the three representative configurations are illustrated in Figures S36–S38, and the Gibbs free‐energy diagrams are provided in Figure 5F. Specifically, the limiting thermodynamic step on PdCuNiCoFe HEA is identified as the adsorption of ^*^OH in the presence of ^*^OCH_2_CH_2_OH, featuring an uphill free energy change of 0.41 eV. In comparison, the limiting thermodynamic step on a‐Pd and c‐Pd is the adsorption of ^*^OH in the presence of ^*^CHOCH_2_OH, with a much higher uphill free energy change of 0.64 and 1.07 eV, respectively. The lower free energy change on the amorphous PdCuNiCoFe HEA benefits the selective oxidation pathway of EG to GA and indicates a balanced interaction strength between intermediates and the HEA surface, consistent with optimal catalytic performance as described by the Sabatier principle. In addition, a free‐energy diagram of the C─C bond cleavage pathway is presented in Figure S39, which indicates that the C─C bond cleavage pathway is thermodynamically less favorable on the HEA surface compared to GA formation, as evidenced by the highest uphill free energy difference of 2.68 eV.

## 3 Conclusion

In this work, we demonstrated a versatile method for the simultaneous engineering of the crystal phase and morphology of HEA nanomaterials, i.e., amorphous PdCuNiCoFe nanobranches, and successfully synthesized a library of amorphous Pd‐based nanobranches from binary to HEA alloys. We uncovered that Pd and Cu played an important role in defining both the branch morphology and amorphous phase. Time‐dependent synthesis and reaction kinetic study unveiled the morphology evolution from nanoparticles to nanobranches and the compositional progression throughout the synthesis. XPS and XAS analyses proved the electron transfer in the PdCuNiCoFe HEA, enabling the regulation of electronic structure for enhanced electrocatalysis. Benefiting from the amorphous phase and high entropy effect, the PdCuNiCoFe HEA catalyst delivered excellent electrocatalytic performance toward EGOR with a high mass activity (5.8 A mg_Pd_^−1^), exceptional selectivity for GA with a maximum FE of 98.6% that, surpassing a majority of Pd‐based catalysts in recent studies. When integrated into an AEMWE for EGOR‐assisted water electrolysis, the amorphous HEA nanobranch catalyst displayed a substantially decreased overpotential compared with overall water electrolysis, delivering an industrial‐level current density (500 mA cm^−2^) at 1.66 V and a long durability for 300 h. In situ characterizations validate the notable suppression of C‒C cleavage on the amorphous PdCuNiCoFe HEA than on the commercial Pd/C, thus favoring the selective oxidation of EG into value‐added GA. DFT calculations further demonstrated the optimized electronic structure, favorable adsorption ability of reaction intermediates, and reduced free energy change during EGOR within the PdCuNiCoFe HEA. This work develops an appealing route to simultaneously engineering both the morphology and crystal phase of HEA nanomaterials, offering an efficient class of HEA catalysts for enhanced electrocatalysis and necessary insights into the structure‐performance relation of HEA catalysis.

## Author Contributions

B.H. and M.Z. conceived the project. B.H. conducted the synthesis and electrochemical experiments. B.H., M.B., D.F., and C.E. contributed to sample characterizations. J.L. and J.C. carried out the in situ characterizations. F. F. conducted the EPR test. X.C. performed DFT simulations. B.H., X.C., and M.Z. conducted data analysis. B.H. and M.Z. analyzed results and wrote the manuscript. M.Z. directed research.

## Conflicts of Interest

The authors declare no conflicts of interest.

## Supporting information




**Supporting File**: adma73384‐sup‐0001‐SuppMat.docx.

## Data Availability

The data that support the findings of this study are available on request from the corresponding author. The data are not publicly available due to privacy or ethical restrictions.
