# Supplementary material for: Simultaneously Engineering the Amorphous Phase and Branch Morphology of High‐Entropy Alloy Nanomaterials for Enhanced Ethylene Glycol Oxidation
Source: Adv Mater. 2026 May 14;38(34):e73384. doi: 10.1002/adma.73384 (PMC13274695; doi:10.1002/adma.73384)
Supplement: Supplementary file 1 — Supporting File: adma73384‐sup‐0001‐SuppMat.docx. [file ADMA-38-e73384-s001.docx]

**Supporting Information for**

**Simultaneously engineering the amorphous phase and branch morphology of high-entropy alloy nanomaterials for enhanced ethylene glycol oxidation**

*Biao Huang*, *Xinyu Chen*, *Jianbin Luo*, *Jing Cao*, *Deqi Fan*, *Min Bi*, *Fukai Feng*, *Lei Huang*, *Chin Yi En*, *Yu Zhang*, *Zhiheng Lyu*, *Ming Zhao*^*^

B. Huang, X. Chen, J. Luo, J. Cao, D. Fan, M. Bi, L. Huang, C. En, M. Zhao

Department of Materials Science and Engineering, National University of Singapore, Singapore 117575, Singapore

Email: [mingzhao@nus.edu.sg](mailto:mingzhao@nus.edu.sg)

F. Feng

School of Materials Science and Engineering, University of Science and Technology Beijing, Beijing 100083, China

Z. Lyu

Department of Chemical and Biological Engineering, University of Alabama, Tuscaloosa, AL 35487, United States

Y. Zhang

School of Mechanical and Power Engineering, East China University of Science and Technology, Shanghai, 200237, China

L. Huang, M. Zhao

Centre for Hydrogen Innovations, National University of Singapore, Singapore 117580, Singapore

B. Huang, and X. Chen contributed equally to this work.

**Methods**

**Materials**

Sodium tetrachloropalladate(II) (Na_2_PdCl_4_, ≥99.99% trace metals basis), copper(II) chloride dihydrate (CuCl_2_·2H_2_O, reagent grade), ascorbic acid (AA, ≥99%), and iron(III) chloride (FeCl_3_, analytic grade) were purchased from Sigma-Aldrich. Nickel(II) chloride hexahydrate (NiCl_2_·6H_2_O, analytic grade), cobalt(II) chloride hexahydrate (CoCl_2_·6H_2_O, analytic grade), KOH (electronic Grade), and oleylamine (OAm, 80-90%) were purchased from Shanghai Aladdin Biochemical Technology Co., Ltd. All chemicals were used as received without further treatment. All aqueous solutions were prepared using deionized water with a resistivity of 18.2 MΩ cm at room temperature.

**Synthesis of** **amorphous PdCuNiCoFe high entropy alloy (HEA) nanobranches and other alloys**

In a standard synthesis, Na_2_PdCl_4_ (4 mg), CuCl_2_·2H_2_O (2.6 mg), NiCl_2_·6H_2_O (8.0 mg), CoCl_2_·6H_2_O (1.0 mg), and FeCl_3_ (1.6 mg) were dissolved in OAm (5 mL) and stirred for 3h. Subsequently, Subsequently, 40 mg of AA was introduced into the above solution, and the resulting mixture was stirred for an additional 1 h. The reaction was then maintained at 60 °C in an oil bath for 8 h, after which it was allowed to cool naturally to ambient temperature. The solid product was isolated by centrifugation at 12,000 rpm for 3 min upon the addition of 15 mL of ethanol. Finally, the as-prepared amorphous PdCuNiCoFe high-entropy alloy was re-dispersed in toluene for subsequent applications. Amorphous Pd-based alloys, including PdCu, PdCuNi, PdCuCo, PdCuFe, PdCuNiCo, and PdCuNiFe, were prepared following the standard synthesis except for adding the corresponding metal precursors. For example, Na_2_PdCl_4_ (4 mg) and CuCl_2_·2H_2_O (2.6 mg) were added for the synthesis of amorphous PdCu.

**Characterizations**

Transmission electron microscopy (TEM) images and selected area electron diffraction (SAED) patterns were taken on the JEOL 2010F. The high-angle annular dark-field scanning TEM (HAADF-STEM) images and energy dispersive spectroscopy (EDS) results were taken on a FEI Themis Z spherical aberration-corrected transmission electron microscope. X-ray photoelectron spectroscopy (XPS) characterization was conducted using a Thermo ESCALAB 250Xi apparatus. The binding energy was calibrated by assigning the C 1s peak to 284.8 eV. Elemental concentrations were determined by inductively coupled plasma optical emission spectroscopy (ICP-OES) employing a Thermo iCAP PRO XP system. X-ray Diffraction (XRD) patterns were collected on a Bruker D8 Advance X-ray diffractometer with Cu-Kα radiation. X-ray absorption spectroscopy (XAS) at the Pd K-edges was collected in transmission mode at the Shanghai Synchrotron Radiation Facility (SSRF). The electron paramagnetic resonance (EPR) measurement was conducted using an Endor spectrometer (Bruker E500) at room temperature.

**Electrochemical measurements**

Electrochemical measurements were conducted in a three-electrode system at room temperature using a Cortest CS310X electrochemical workstation. A Hg/HgO electrode and a graphite rod were used as the reference and the counter electrode, respectively. The catalyst was loaded on the carbon support (Vulcan XC-72) first and then mixed with ethanol and Nafion alcohol solution (5 wt%) to form a homogeneous ink. Subsequently, catalyst ink containing 30 μg of Pd was drop casted to the carbon paper electrode (0.5 cm^2^) to serve as the working electrode. All potentials were converted into values in reference to reversible hydrogen electrode (RHE) according to *E*(RHE) = *E*(Hg/HgO) + 0.097 + 0.059 × pH.

Cyclic voltammetry (CV) measurements were first carried out at 50 mV·s^−1^ for 50 cycles to get stable CV curves, in N_2_-saturated 1.0 M KOH solution. The electrochemically active surface areas (ECSAs) were calculated based on the charge required for oxygen desorption, that is, from the area of the reduction peak of PdO in the as-obtained CV curves. Then, the activity of the catalysts towards ethylene glycol oxidation reaction (EGOR) was evaluated by CV measurements in a mixed N_2_-saturated solution containing 1.0 M KOH and 1.0 M EG at 50 mV·s^−1^. Linear sweep voltammetry (LSV) curves were recorded at a scan rate of 10 mV s^−1^ with 95% iR (ohmic) compensation. The stability of the catalysts was evaluated by chronoamperometric measurements at 0.82 V for 36,000 s in the mixed solution of 1.0 M KOH and 1.0 M EG.

**Product analysis**

The ^1^H nuclear magnetic resonance (NMR) measurements were recorded on a 300 MHz Bruker spectrometer (Bruker Avance III HD, BBO Probe). For the NMR test, 600 μL of electrolyte was added with 30 μL of a mixed solution of D_2_O and dimethyl sulfoxide (DMSO), in which DMSO was used as the internal standard. For the analysis of the product distribution after EGOR in the three-electrode system, the electrolyte solution was collected after long-term chronoamperometric measurements to accumulate enough products. Freshly prepared electrolytes were used for tests under different applied potentials. Then, the electrolyte was subjected to the NMR test. The concentration of products in the electrolyte solution was calculated by using a standard curve showing the relationship between the integral peak area in NMR spectra and the concentration of products. Then, FE was determined using the following equation: FE = [(*Z*·*n*·*F*)/*Q*] × 100%, where *Z* is the number of electrons required to produce a given product, *n* (mol) is the number of moles of the product, *F* (96485 C mol^−1^) is the Faraday constant, and *Q* (C) is the total charge released.

**Anion exchange membrane water electrolyzer (AEMWE) performance evaluation**

The membrane-electrode-assembly (MEA)-based performance of an AEMWE device was evaluated using a commercially available test station (Figure S23). The anode and cathode were fabricated by depositing the Amorphous PdCuNiCoFe HEA and commercial Pt/C catalyst ink onto the Ni foam, respectively. A Fumasep FAA-3-PK-130 anion exchange membrane was sandwiched between the cathode and anode. The AEMWE electrolyzer was operated at a temperature of 80 °C under an ambient pressure. Polarization curves were obtained to evaluate the performance, and chronoamperometric measurements tests were conducted to probe the long-term stability of the device.

***In situ* characterization**

*In situ* attenuated total reflection surface-enhanced infrared absorption spectroscopy (ATR-SEIRAS) spectroscopy results were collected on a Nicolet iS50 FT-IR spectrometer equipped with a mercury cadmium telluride (MCT) detector. The Au-coated Si hemispherical prism (20 mm in diameter, MTI Corporation) was used as the conductive substrate and the IR reflection element. Background spectra were taken at open-circuit potential and subtracted from each measurement. *In situ* Raman results were collected on a DXR3 Raman Microscope with a 633 nm excitation wavelength laser.

**Density functional theory (DFT) calculations**

All first-principles calculations were performed using DFT in the Vienna ab initio Simulation Package (VASP)^1^. The interaction between valence electrons and ionic cores was described using the projector augmented wave (PAW) method^2^. Exchange-correlation effects were treated within the generalized gradient approximation using the Perdew-Burke-Ernzerhof (PBE) functional^3^. A plane-wave kinetic energy cutoff of 450 eV was employed for all calculations. Brillouin zone sampling was conducted using a Monkhorst-Pack k-point mesh of 3 × 3 × 1. The electronic self-consistency convergence criterion was set to 10^−5^ eV. All atomic positions were fully relaxed until the residual forces on each atom were less than 0.01 eV Å^−1^. All calculations were carried out with spin polarization.

For the crystalline Pd (c-Pd) surface model, the Pd(100) facet was selected due to its high catalytic activity in relevant reactions^4, 5^. The c-Pd surface was modeled using a three-layer slab with three atomic layers of Pd. A vacuum layer with a thickness of 20 Å was included to prevent interactions between periodic images of the surface. During structural relaxation, all atoms in the three-layer Pd slab were allowed to relax. However, in the adsorption calculations, the bottom two layers of the Pd slab were fixed to mimic the bulk environment, while the top layer (the surface layer) was allowed to relax to optimize the surface structure. For the amorphous Pd (a-Pd) and amorphous HEA models, we used the similar simulation parameters as those for the crystalline Pd surface (Table S3), ensuring sufficient Pd exposure on the surface. This approach reflects the disordered nature of the material and allows for a comparison with the crystalline Pd surface, providing insight into the differences between the ordered and disordered structures.

The amorphous PdCuNiCoFe HEA structure was constructed using ab initio molecular dynamics (AIMD) simulations, following a typical melt-and-quench approach^6^. The process began with a crystalline Pd model, which was annealed at 1,000 K to form amorphous Pd. The initial melting temperature was set to 1,000 K, followed by an equilibration period of 5 ps to ensure thermal stability before quenching. The quenching rate was precisely set to 40 K/ps to control the cooling process and allow the formation of a stable amorphous phase. The total simulation time was 22.5 ps, with a time step of 1 fs to ensure accurate sampling of the system. The simulation was conducted using the NVT ensemble with a Nosé-Hoover thermostat to control the temperature, and the system was allowed to adjust without the use of a barostat, as the simulation was conducted under constant volume. All atomic positions were fully relaxed until the residual forces on each atom were minimized to ensure reliable total energies and optimized geometries. The supercell contained 92 atoms, ensuring a sufficiently large system size for meaningful simulation results. A 2×2×2 k-point mesh was employed during the AIMD simulations to properly sample the Brillouin zone, which differs from static calculations. Afterward, based on the atomic ratios determined experimentally by ICP-OES analysis, four elements, including Cu, Co, Ni, and Fe, were randomly distributed within the Pd matrix. This process was performed in 20 distinct configurations, from which the structure with the lowest energy was selected as the representative model for further studies. In the adsorption calculations, the bottom two layers of the PdCuNiCoFe supercell were fixed, while the top layer was allowed to relax. A radial distribution function (RDF) plot is provided (Figure S32), showing the absence of long-range order.

The adsorption energy (*E*_ads_) was calculated as the energy difference between the adsorbate–surface system and the sum of the energies of the clean surface and the isolated adsorbate molecule.

*E*_ads_​ = *E*_surface+adsorbate​_ − *E*_surface_ _​_− *E*_adsorbate_​

The free energy change for each reaction step was calculated following the computational hydrogen electrode (CHE) framework originally proposed by Nørskov et. al^7^. In this model, the free energy of the proton-electron pair was equated to that of 1/2 H_2_. The involvement of OH⁻ species is treated within the same thermodynamic framework by relating proton–electron pairs to H_2_ and considering the equilibrium relationship between H_2_O, OH⁻, and (H⁺ + e⁻) under alkaline conditions. The free energy diagrams were constructed at U = 0 V vs. RHE, and the free energy change for each step was computed^8^:

Δ*G* = Δ*E* + Δ*E*_ZPE_​ – *T*Δ*S +* Δ*G_U_*

where Δ*E*, Δ*E*_ZPE_​, Δ*S*, and Δ*G_U_* denote electronic energy difference, zero-point energy, entropy contributions, and the contribution of free energy in electrode potential, respectively, *T* is the temperature. Δ*G_U_* = −neU, where n is the number of electrons transferred, and U is the applied electrode potential.

Because the focus is on the intrinsic properties of the solid surface and reactants, all calculations do not consider solvation models. Although this may result in inaccurate absolute values ​​of adsorption energies and free energy diagrams, the relative trends remain comparable.

**Figure S1.** Enlarged TEM image of amorphous HEA nanobranches.

**
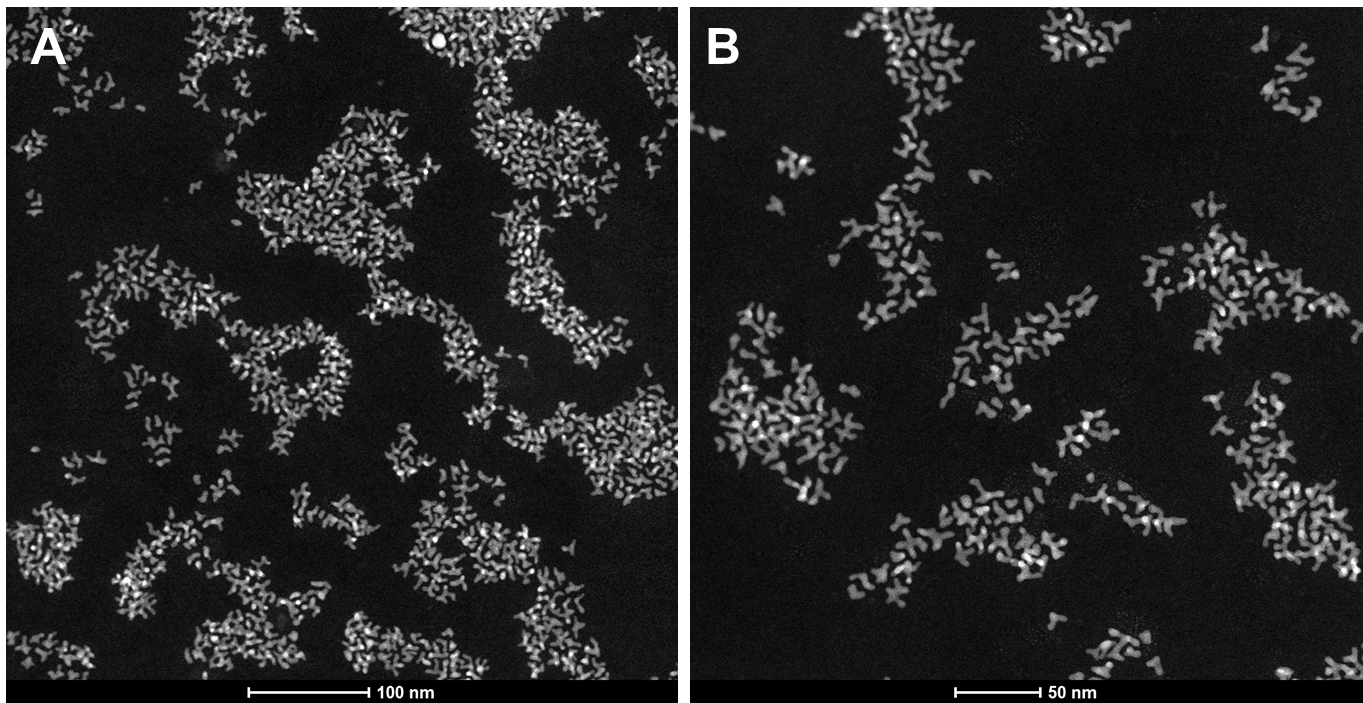
 Figure S2.** (A-B) STEM images of amorphous HEA nanobranches.


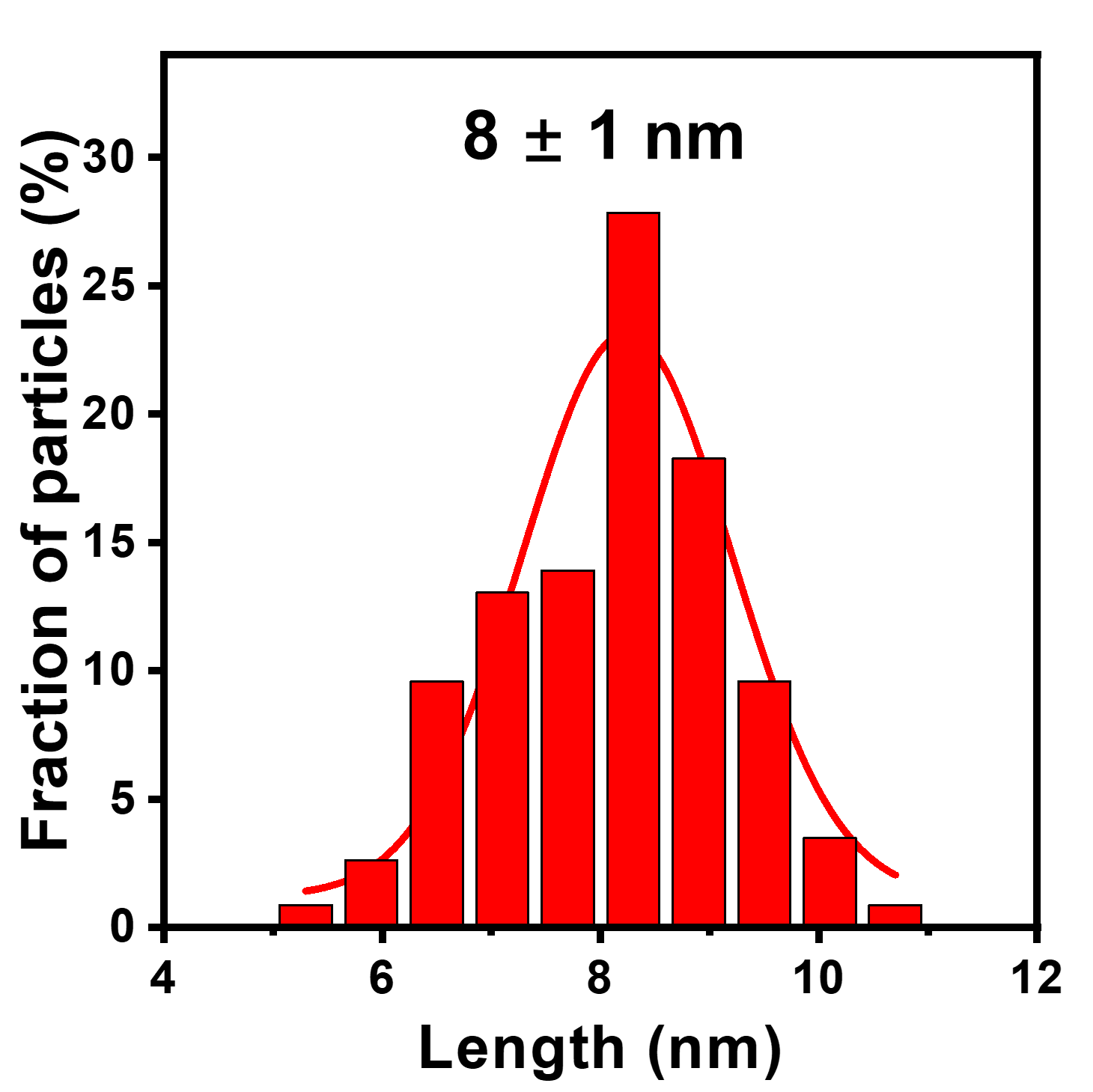


**Figure S3.** Length distribution histogram of the PdCuNiCoFe HEA nanobranches. The value shown in the inset is presented as the mean ± standard deviation.

**Statistical analysis**

The lengths of branches were obtained by averaging over >100 nanoparticles from TEM images. The statistical analyses of particle sizes were performed using the Origin software via Gauss fitting function with the iteration algorithm of Levenberg-Marquardt. The obtained values are presented as the mean ± standard deviation.


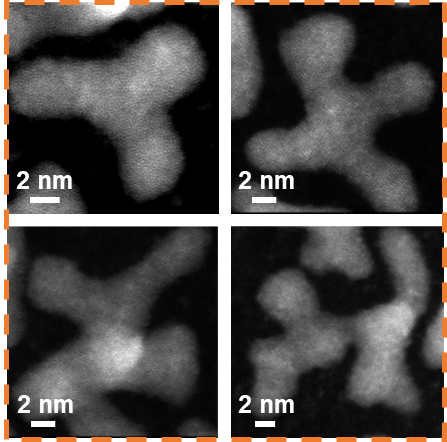


**Figure S4.** HAADF-STEM image of PdCuNiCoFe HEA nanobranches with different numbers of branches.


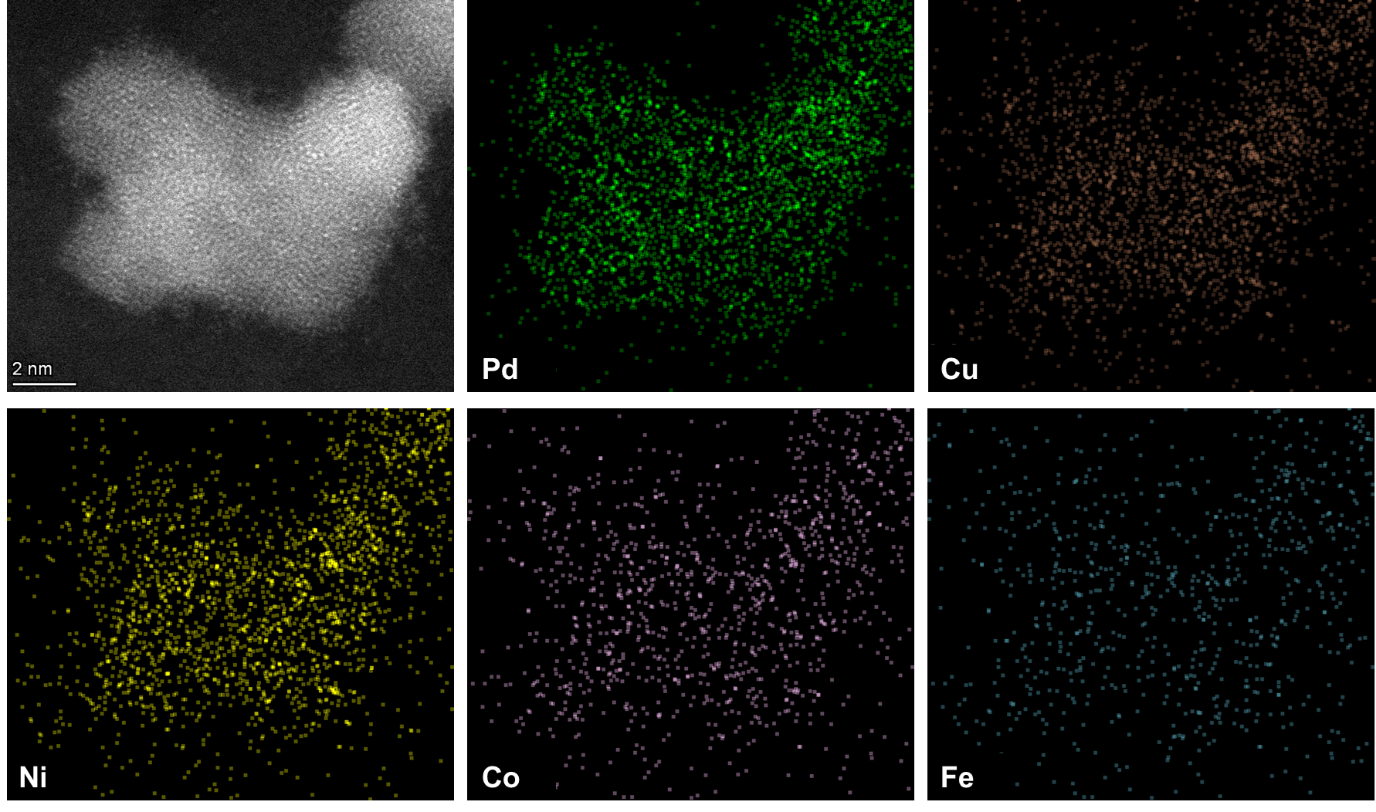


**Figure S5.** HAADF-STEM image and EDS mapping results of PdCuNiCoFe HEA nanobranches.

**
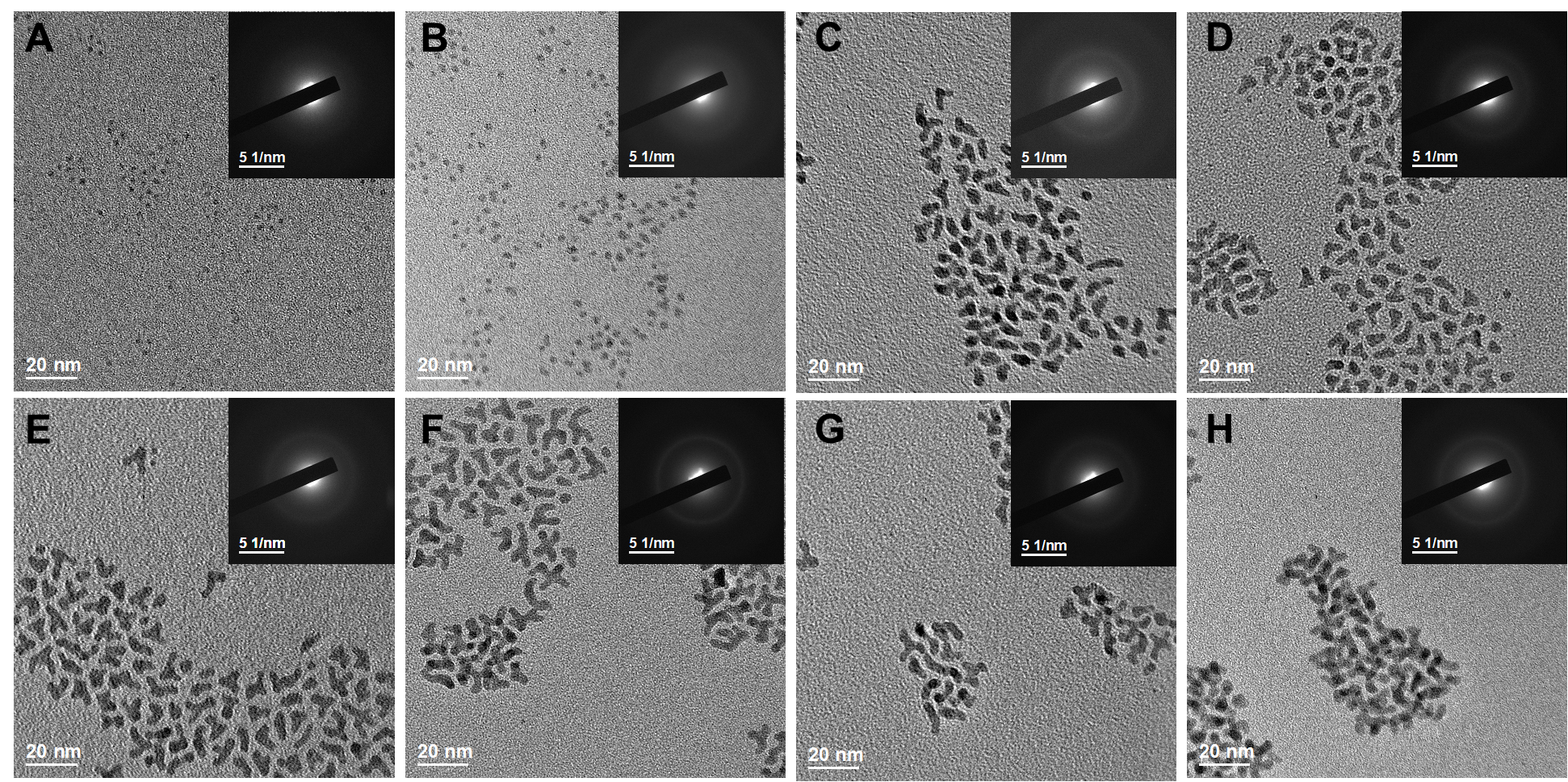
**

**Figure S6.** (A-H) TEM images of the products obtained at different reaction time, *i.e.*, 1h (A), 2h (B), 3h (C), 4h (D), 5h (E), 6h (F), 7h (G), and 8h (H) measured during the time-dependent experiments for the synthesis of the PdCuNiCoFe HEA nanobranches. The inset in each image is the corresponding SAED pattern.


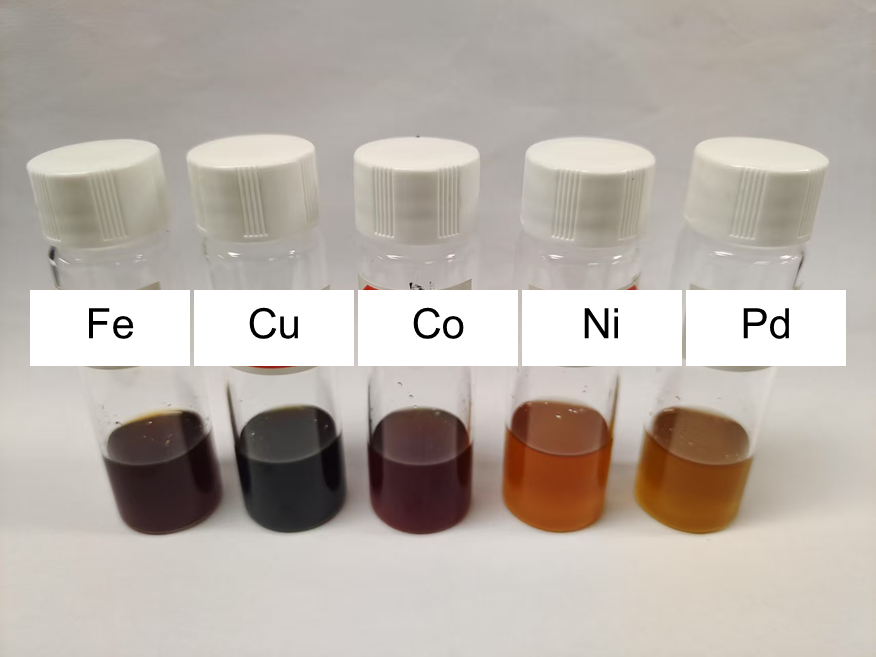


**Figure S7.** Photos of the solution after reaction with different metal precursors added.


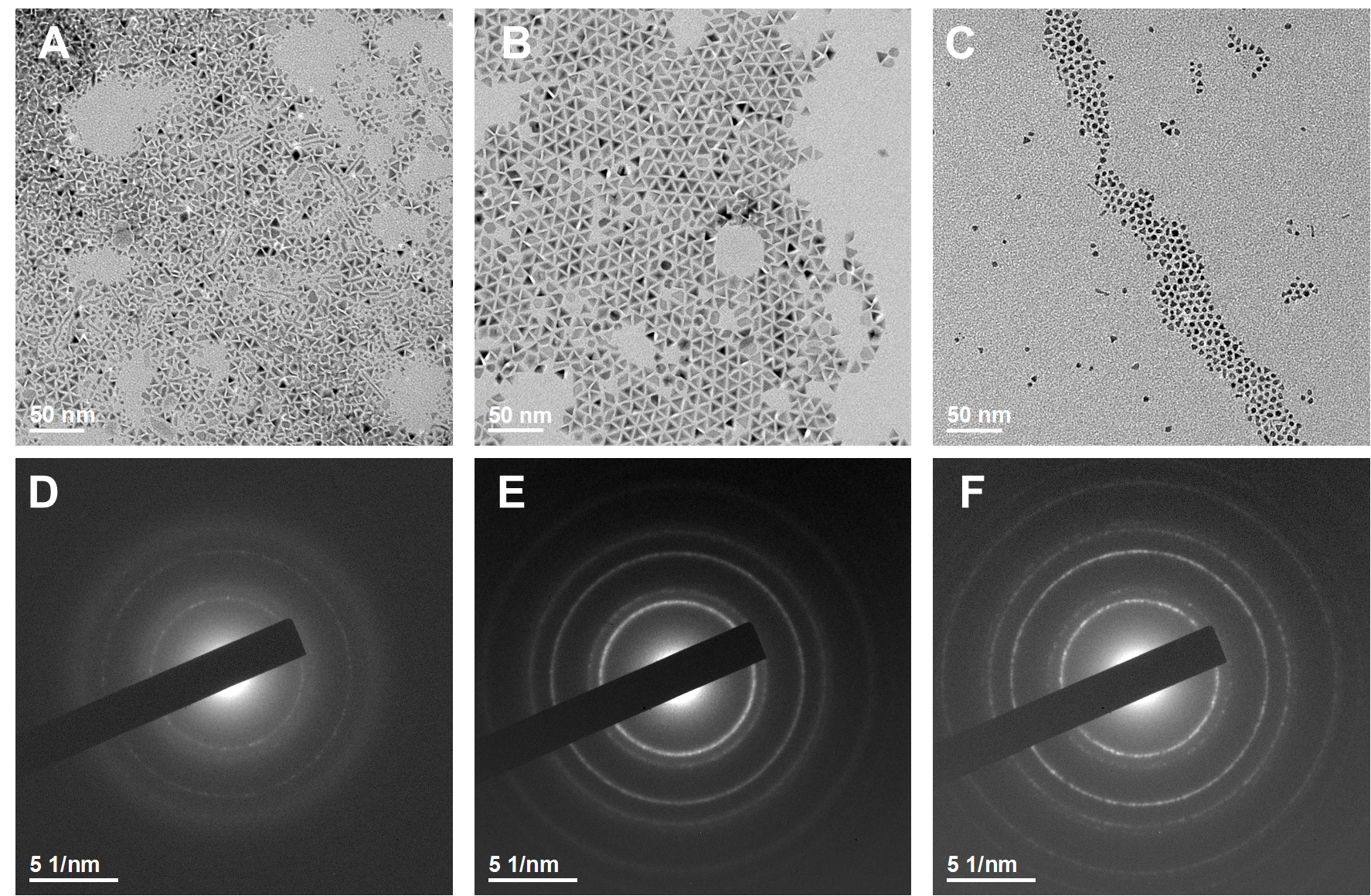


**Figure S8.** TEM images and corresponding SAED patterns of PdNi alloy (A,D), PdCo alloy (B,E), and PdFe alloy (C,F).


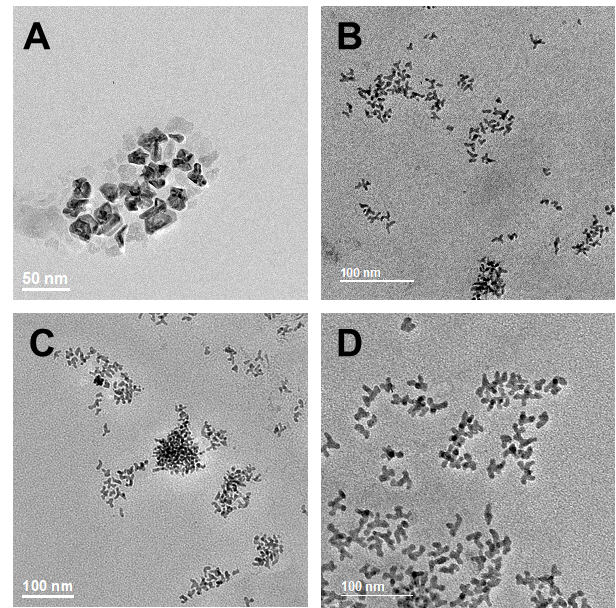


**Figure S9.** (A-D) TEM images of the products during the synthesis of HEA without the addition of Cu precursor (A), Ni precursor (B), Co precursor (C), and Fe precursor (D).


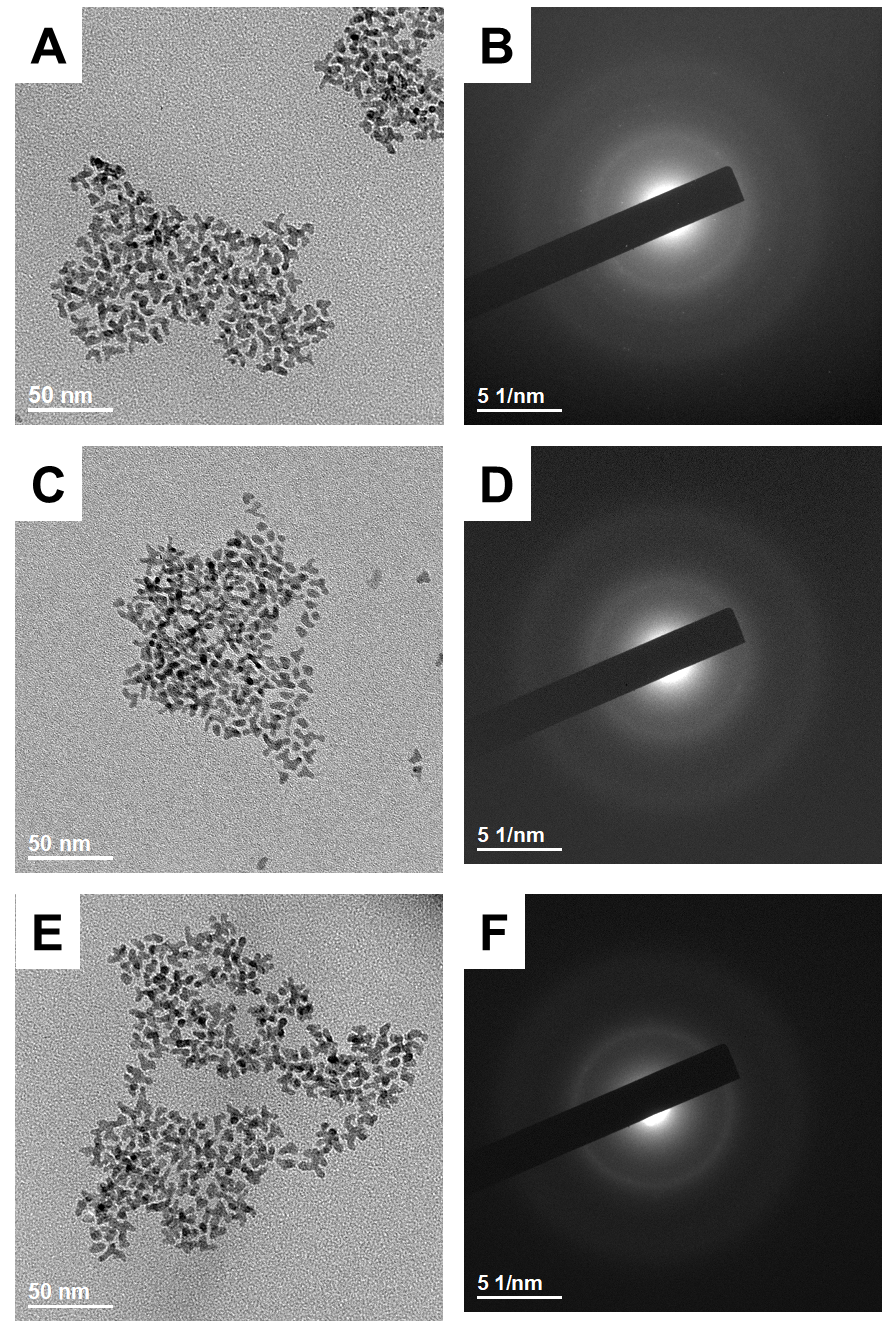


**Figure S10.** (A-F) TEM images and SAED patterns of amorphous nanobranches. (A, C, and E) TEM images of the amorphous PdCuCo (A), PdCuFe (C), and PdCuCoFe (E) nanobranches. (B, D, and F) SAED pattern of the amorphous PdCuCo (B), PdCuFe (D), and PdCuCoFe (F).


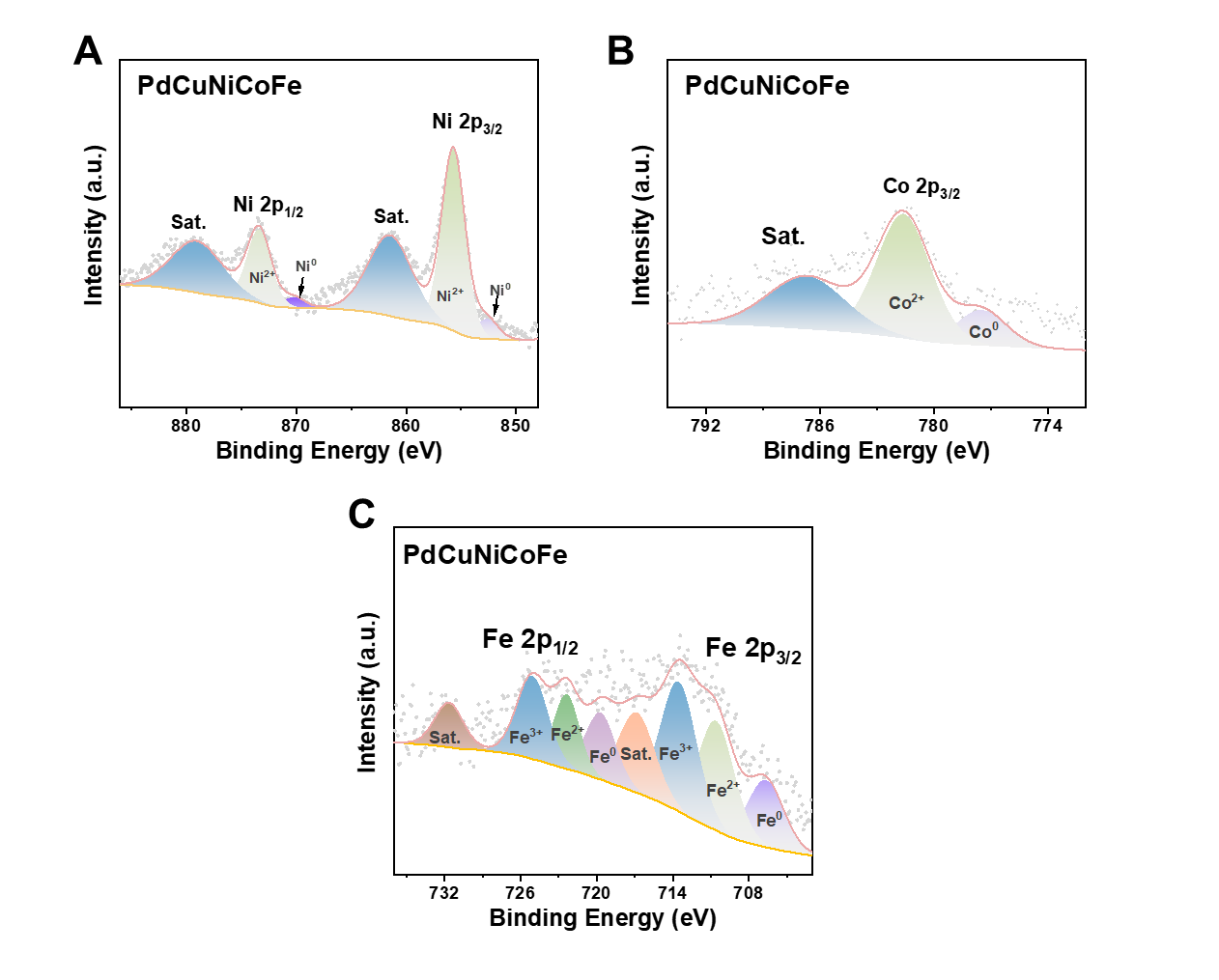


**Figure S11.** (A) Ni 2p, (B) Co 2p, and (C) Fe 2p XPS spectra of the PdCuNiCoFe HEA.


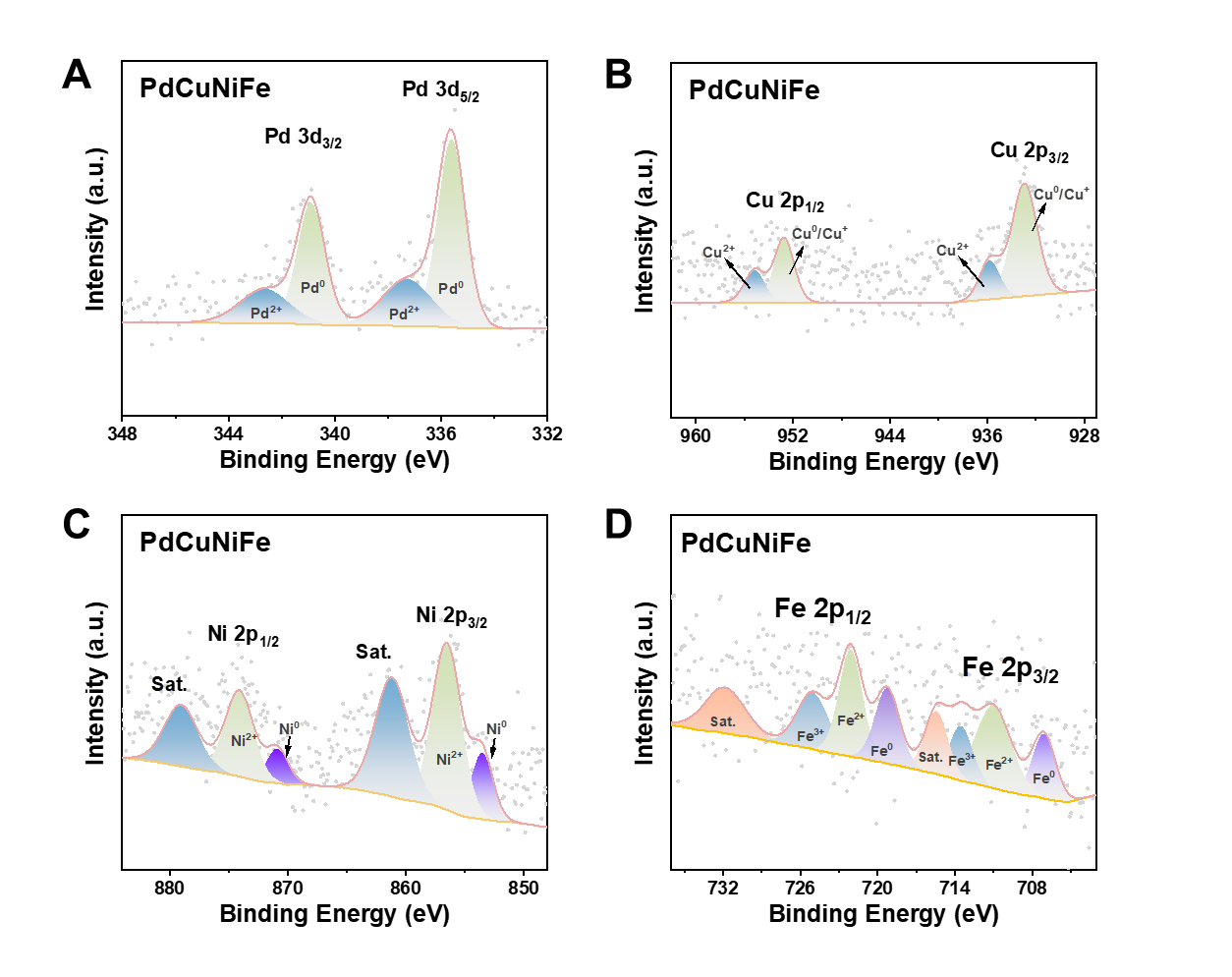


**Figure S12.** (A) Pd 3d, (B) Cu 2p, (C) Ni 2p, and (D) Fe 2p XPS spectra of the PdCuNiFe alloy.


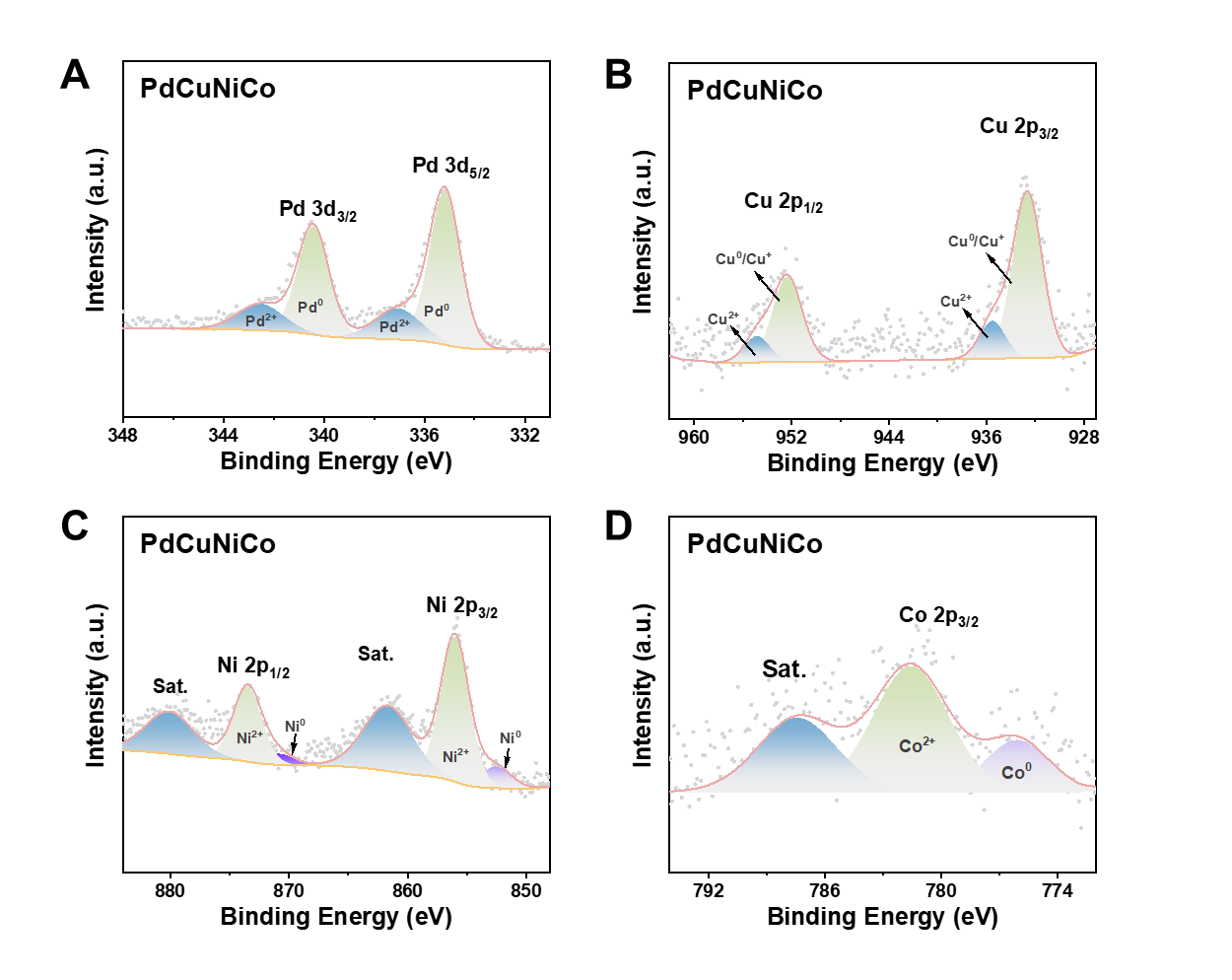


**Figure S13.** (A) Pd 3d, (B) Cu 2p, (C) Ni 2p, and (D) Co 2p XPS spectra of the PdCuNiCo alloy.


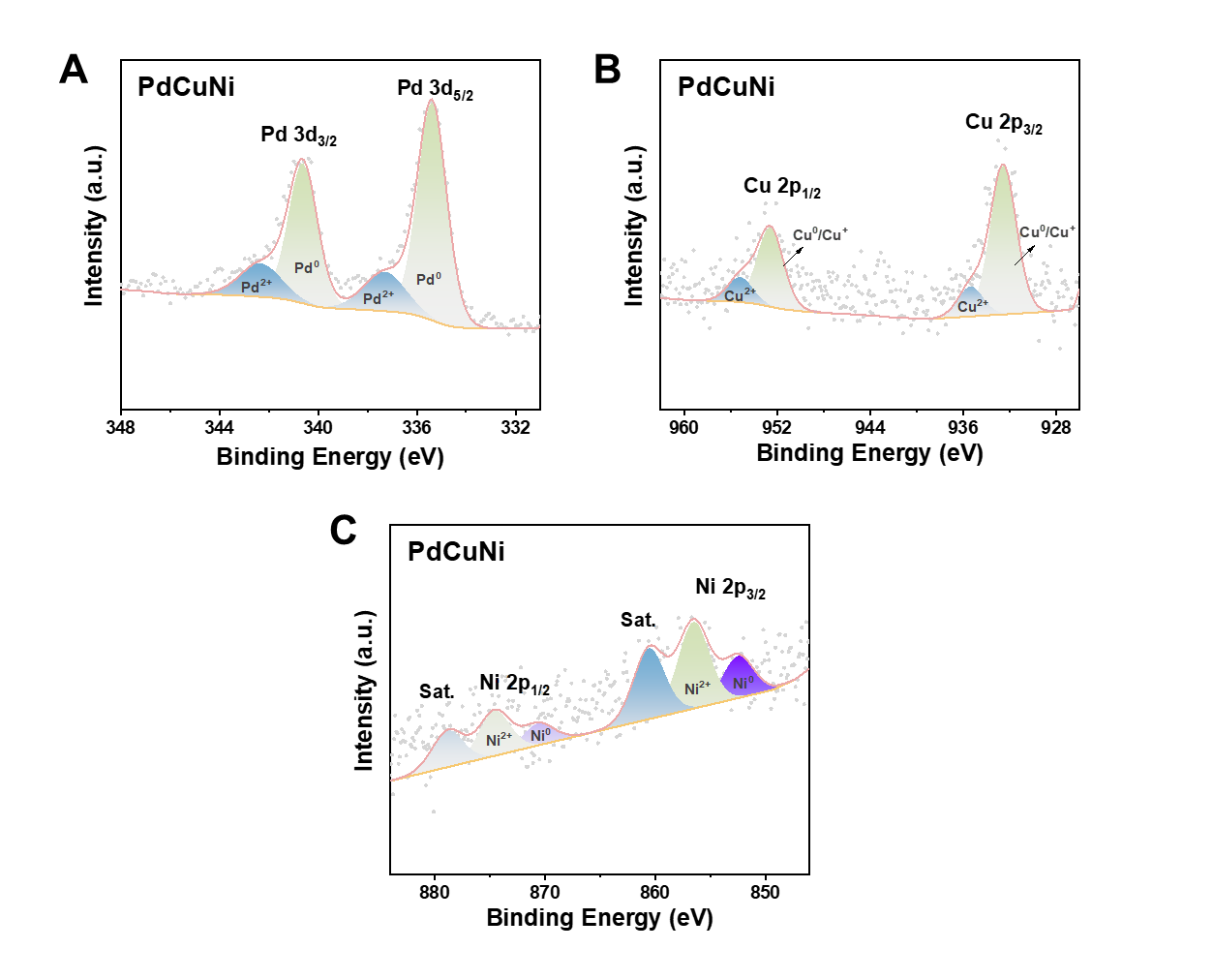


**Figure S14.** (A) Pd 3d, (B) Cu 2p, and (C) Ni 2p XPS spectra of the PdCuNi alloy.


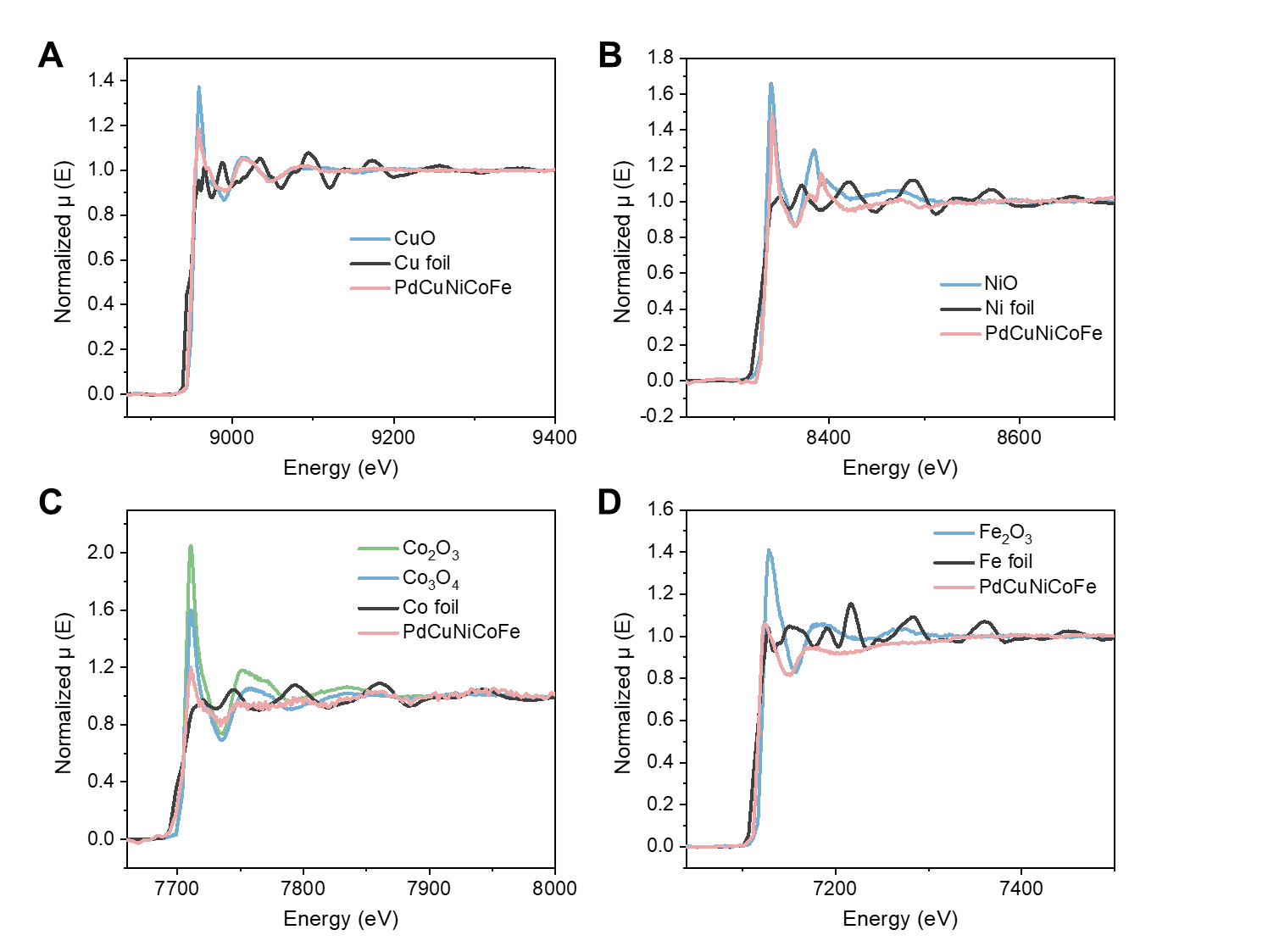


**Figure S15.** *K*-edge XANES spectra of (A) Cu, (B) Ni, (C) Co, and (D) Fe in amorphous PdCuNiCoFe HEA nanobranches.

**Figure S16.** TEM image of the commercial Pd/C catalyst.

**Figure S17.** CV curves recorded in N_2_-saturated 1.0 M KOH aqueous solution at a scan rate of 50 mV·s^−1^.


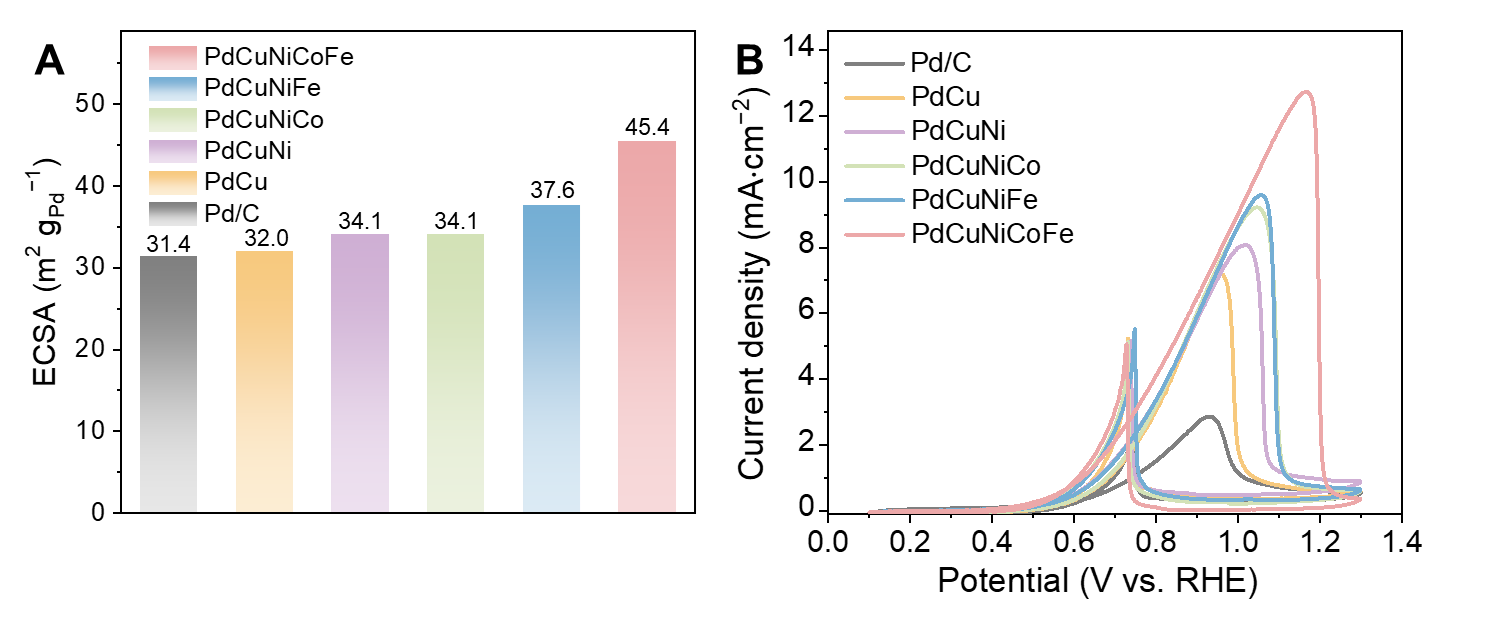


**Figure S18**. (A) The calculated ECSAs of the catalysts based on the corresponding CV curves in Figure S11. (B) ECSA-normalized CV curves of the catalysts in N_2_-saturated aqueous solution containing 1.0 M KOH and 1.0 M EG at a scan rate of 50 mV·s^−1^.

The ECSA (m^2^·g_Pd_^−1^) values of various catalysts were estimated based on ECSA = *Q*/(0.405 × *m*_Pd_), where *Q* is the coulombic charge by integrating peak areas of the reduction of PdO (mC), *m*_Pd_ is the Pd loading (mg·cm^−2^), and the constant (0.405) represents the charge required for the reduction of PdO monolayer (mC·cm^−2^_Pd_).

**
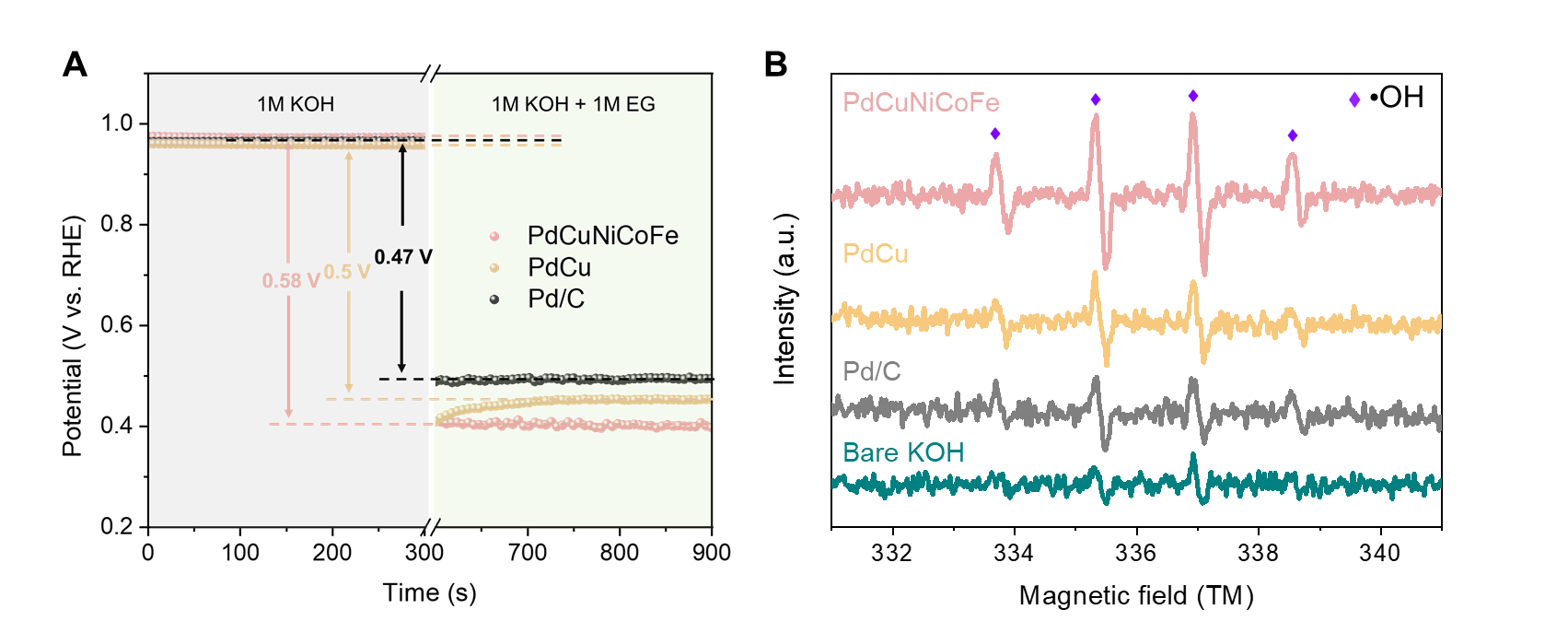
**

**Figure S19**. (A) OCP curves of the amorphous PdCuNiCoFe HEA, amorphous PdCu, and commercial Pd/C in the 1 M KOH electrolyte before and after the injection of 1 M EG. (B) EPR spectra of the electrolytes recorded with DMPO as a spin-trapping agent in the 1 M KOH electrolyte.


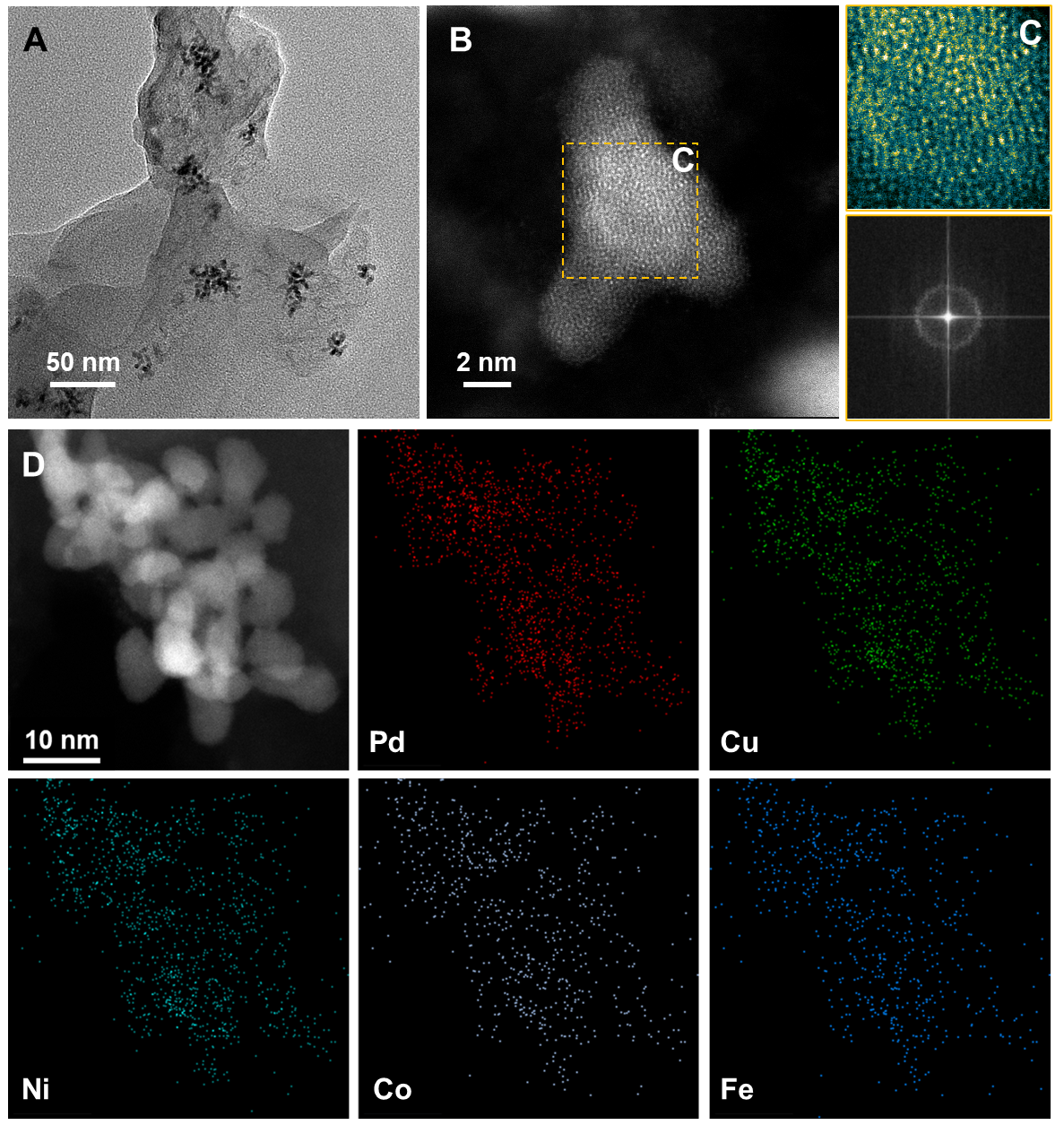


**Figure S20**. (A) TEM image, (B) HAADF-STEM, (C) enlarged HAADF-STEM image with the corresponding FFT pattern of the areas marked in (B), and (D) STEM image with the corresponding EDS elemental mappings of the HEA catalyst after the durability test for EGOR.


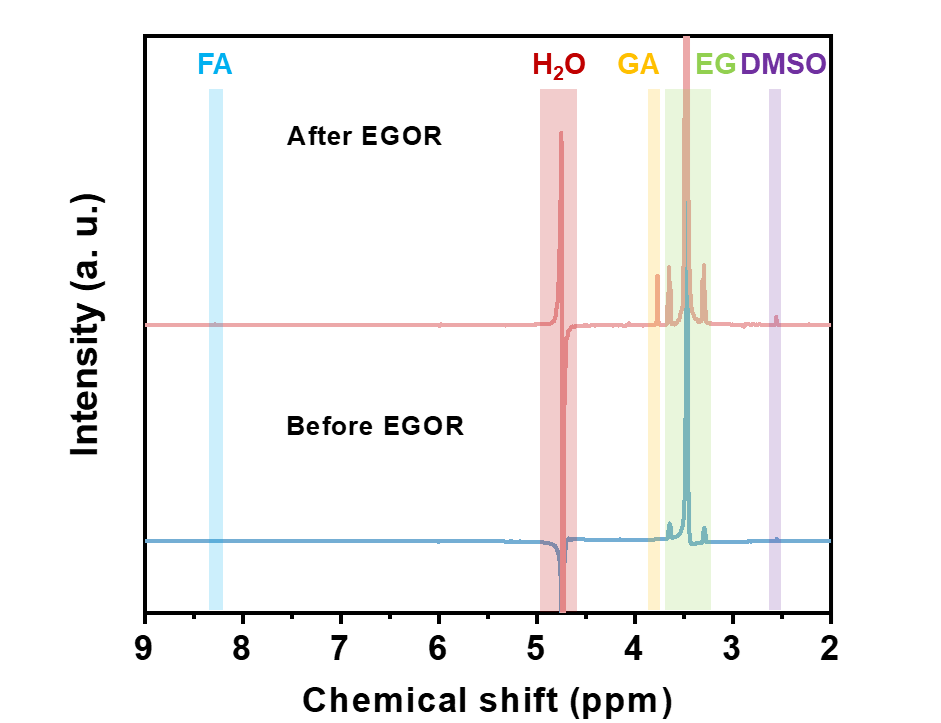


**Figure S21.** ^1^H NMR measurements of the electrolyte before and after the EGOR process on the HEA catalyst. DMSO was added as an internal standard. The water peak at around 4.7 ppm is suppressed with presaturation.

**Figure S22.** Standard curve of GA showing the linear relationship between the integral peak area obtained from NMR and the concentration of GA.

**Figure S23.** Yield rate of GA under different potentials on the HEA catalyst.

**Figure S24.** LSV curves of the catalysts for EGOR in N_2_-saturated 1 M KOH electrolyte with 1 M EG.


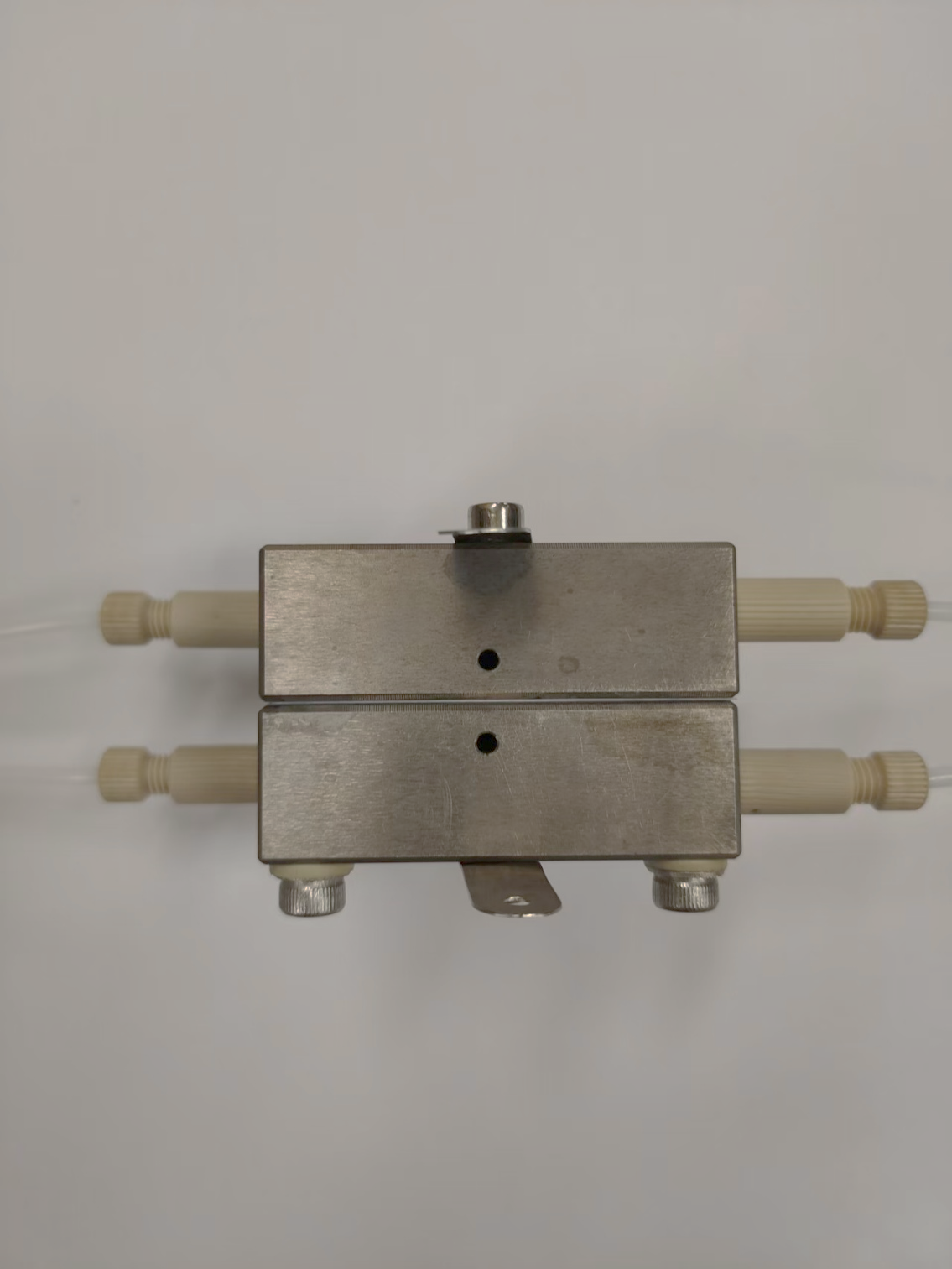


**Figure S25.** Photos of the as-assembled PdCuNiCoFe HEA || Pt/C AEMWE device.

**
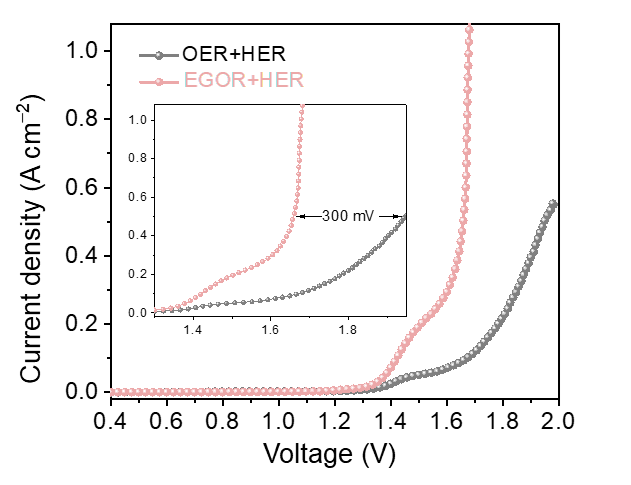
**

**Figure S26.** LSV curves of the PdCuNiCoFe HEA || Pt/C MEA-based AEMWE device with and without 1 M EG.


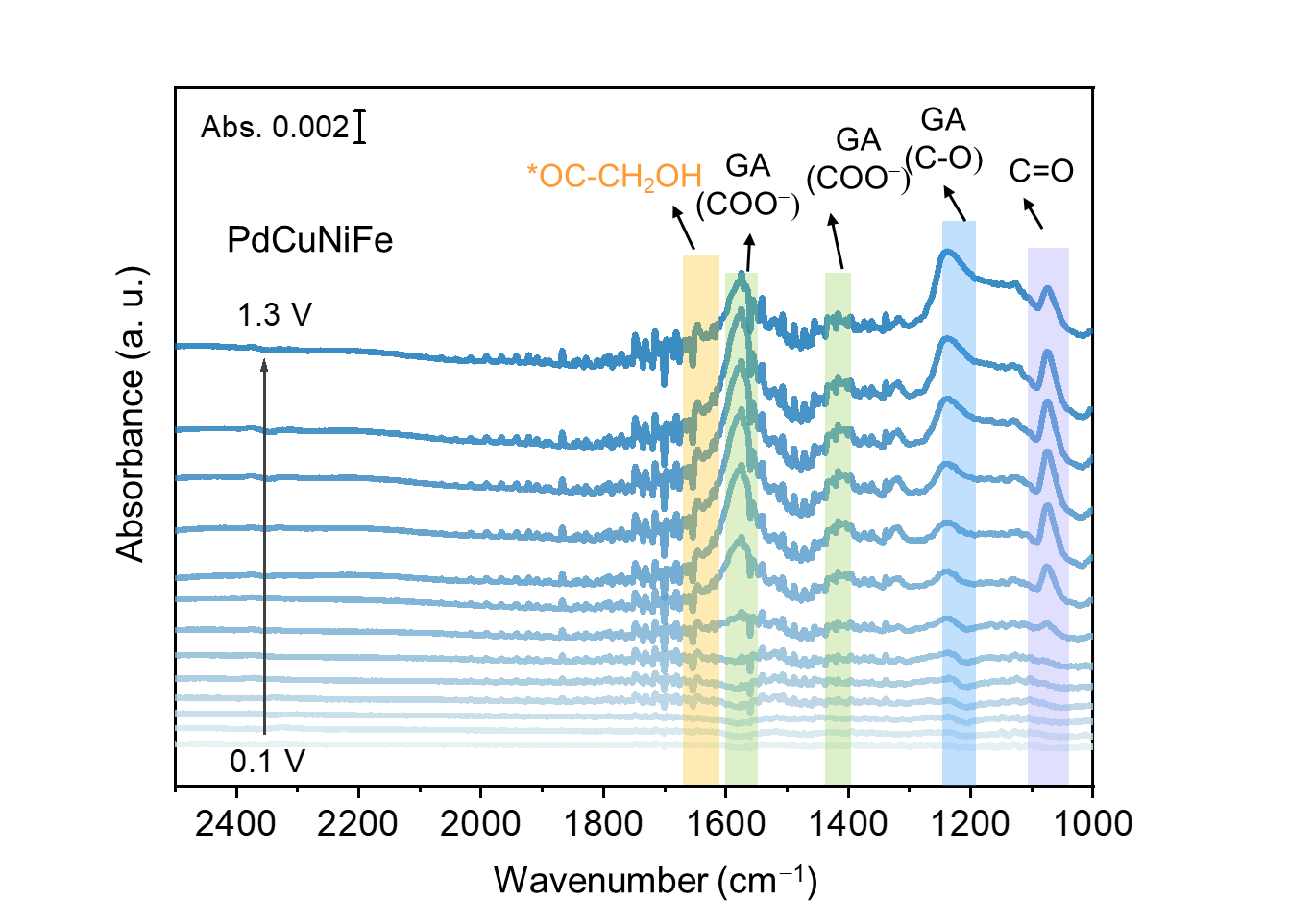


**Figure S27.** ATR-SEIRAS spectra recorded during the electrochemical EGOR on the PdCuNiFe catalysts.


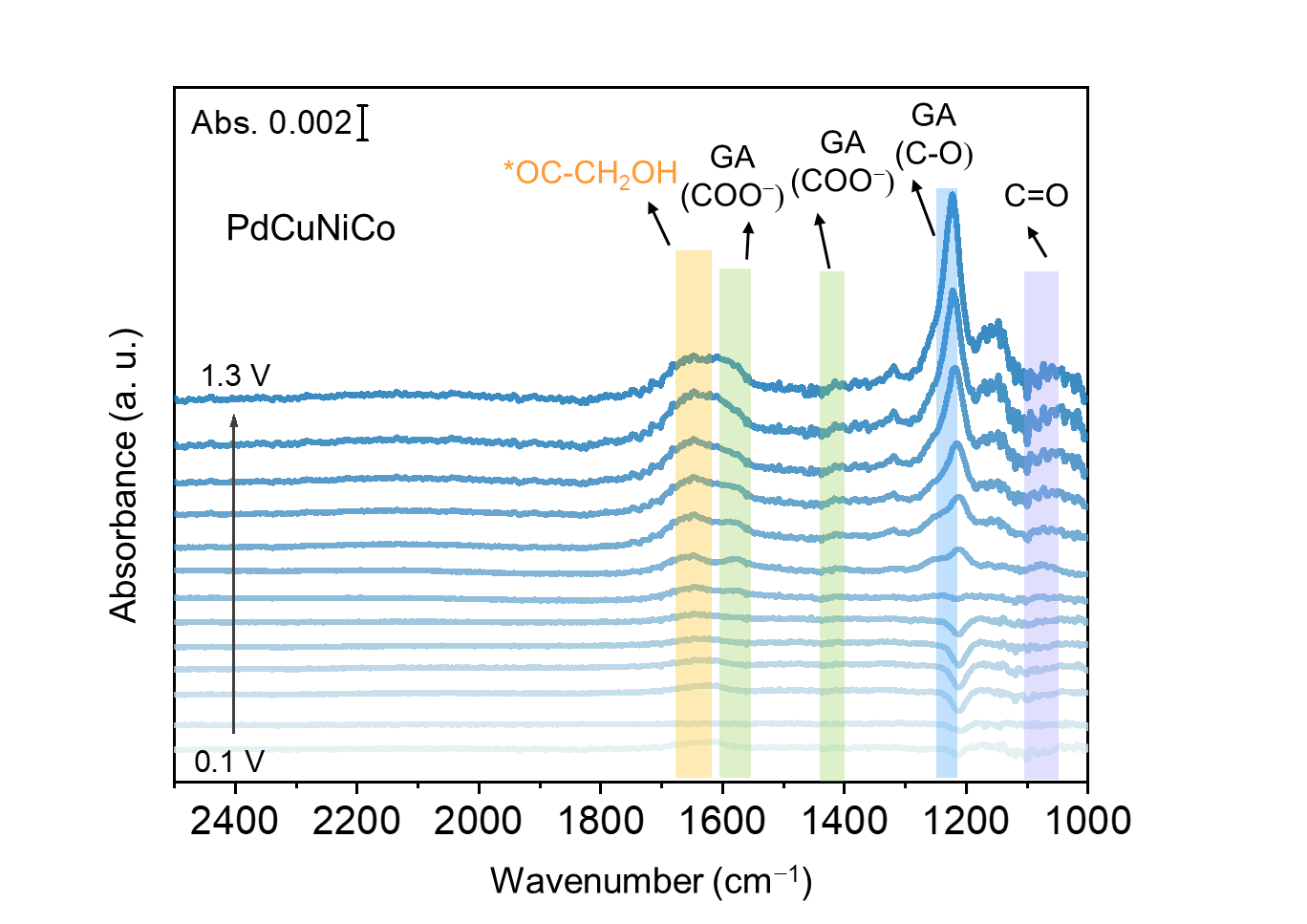


**Figure S28.** ATR-SEIRAS spectra recorded during the electrochemical EGOR on the PdCuNiCo catalysts.


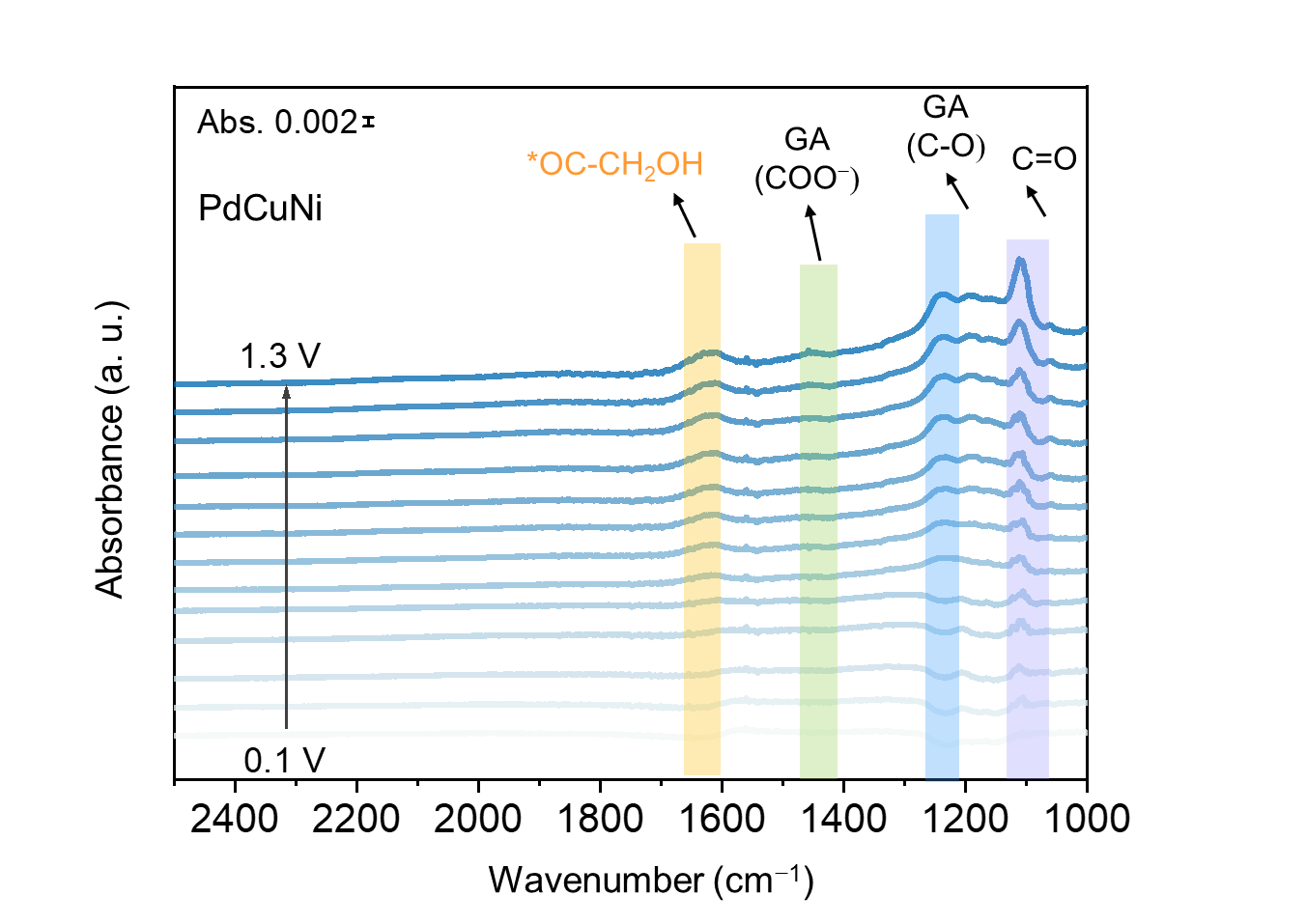


**Figure S29.** ATR-SEIRAS spectra recorded during the electrochemical EGOR on the PdCuNi catalysts.


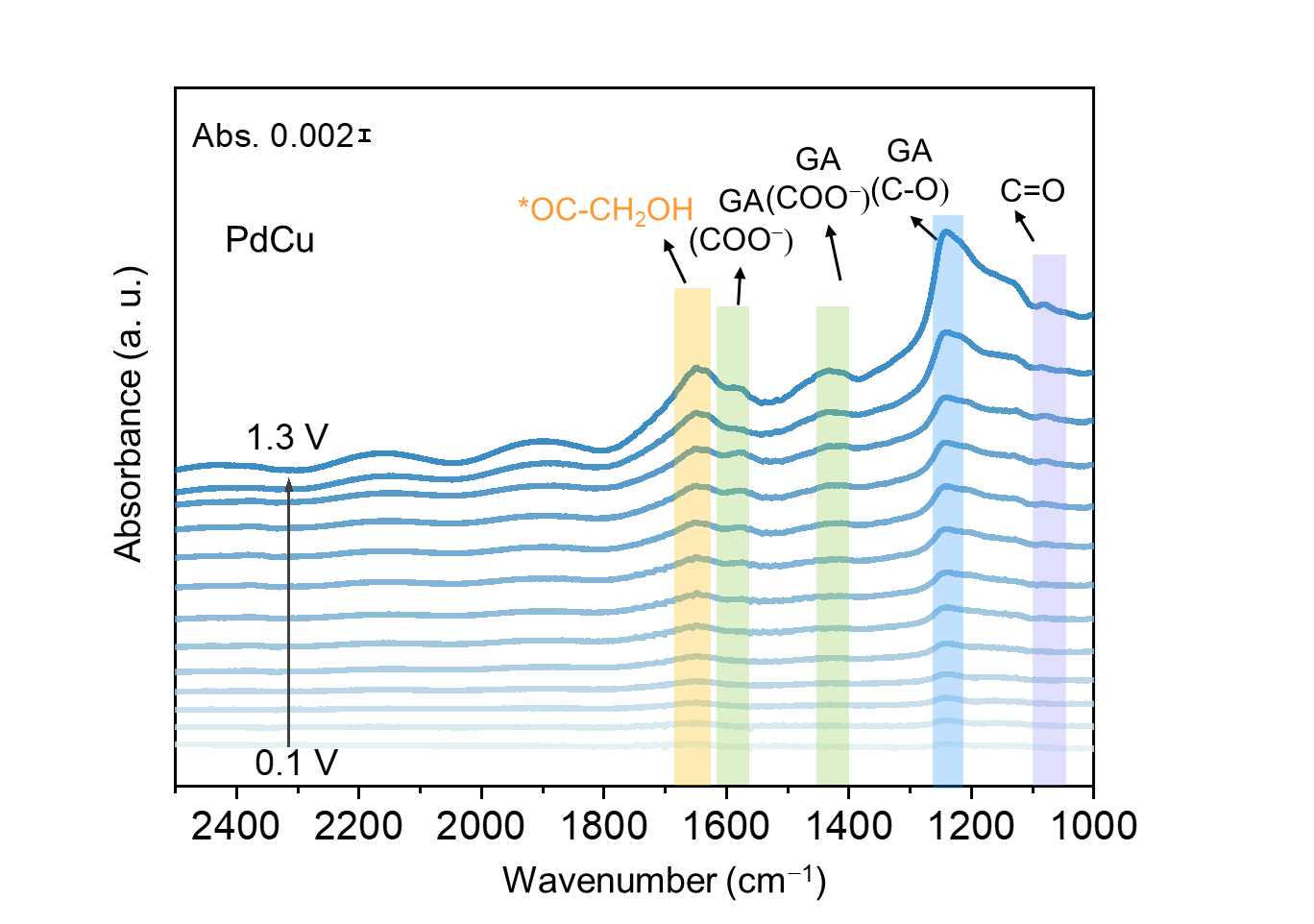


**Figure S30.** ATR-SEIRAS spectra recorded during the electrochemical EGOR on the PdCu catalysts.


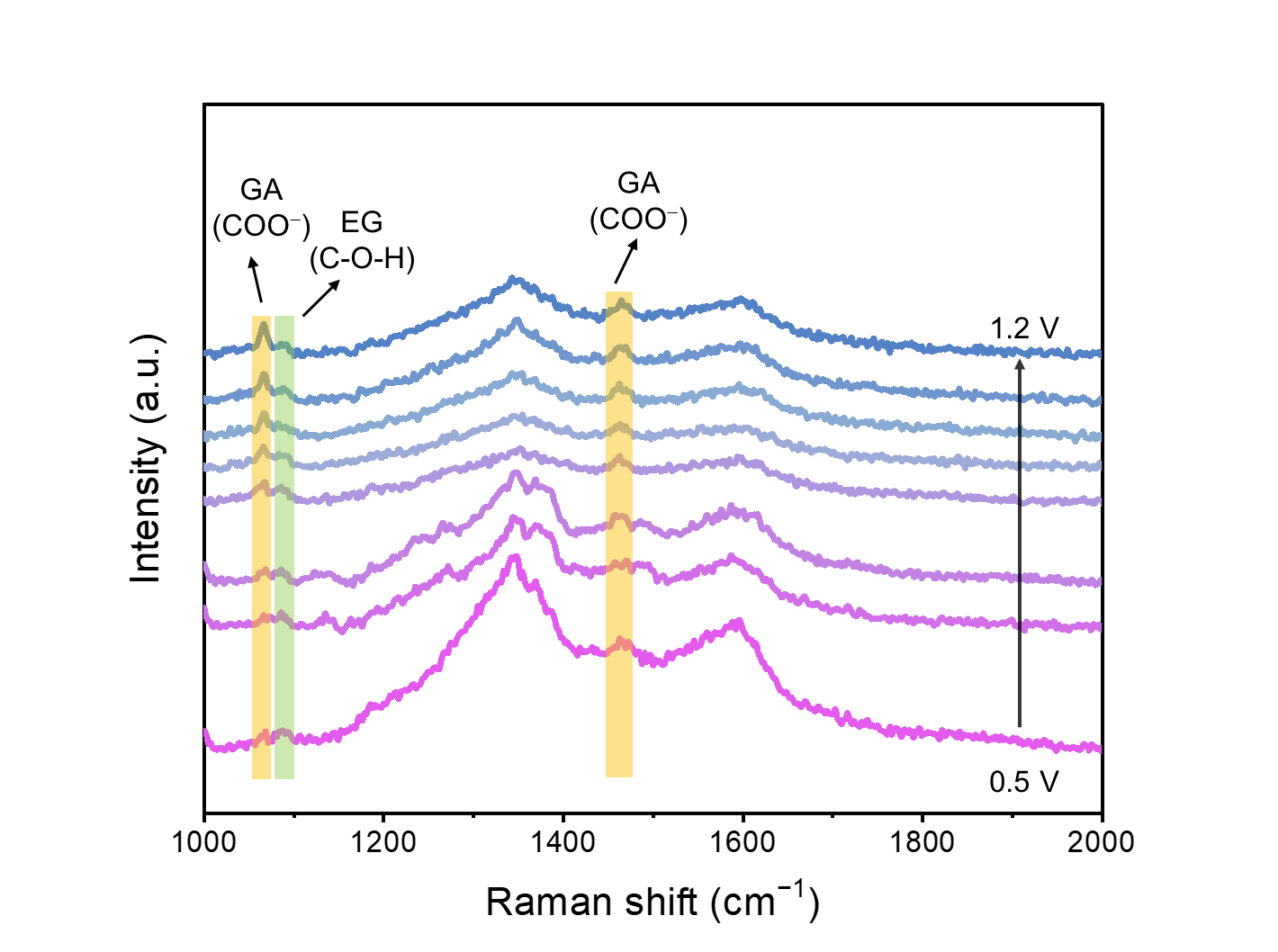


**Figure S31.** *In situ* Raman spectra recorded during the electrochemical EGOR on the PdCuNiCoFe HEA catalysts.

**Figure S32**. RDFs of amorphous Pd and crystalline Pd.


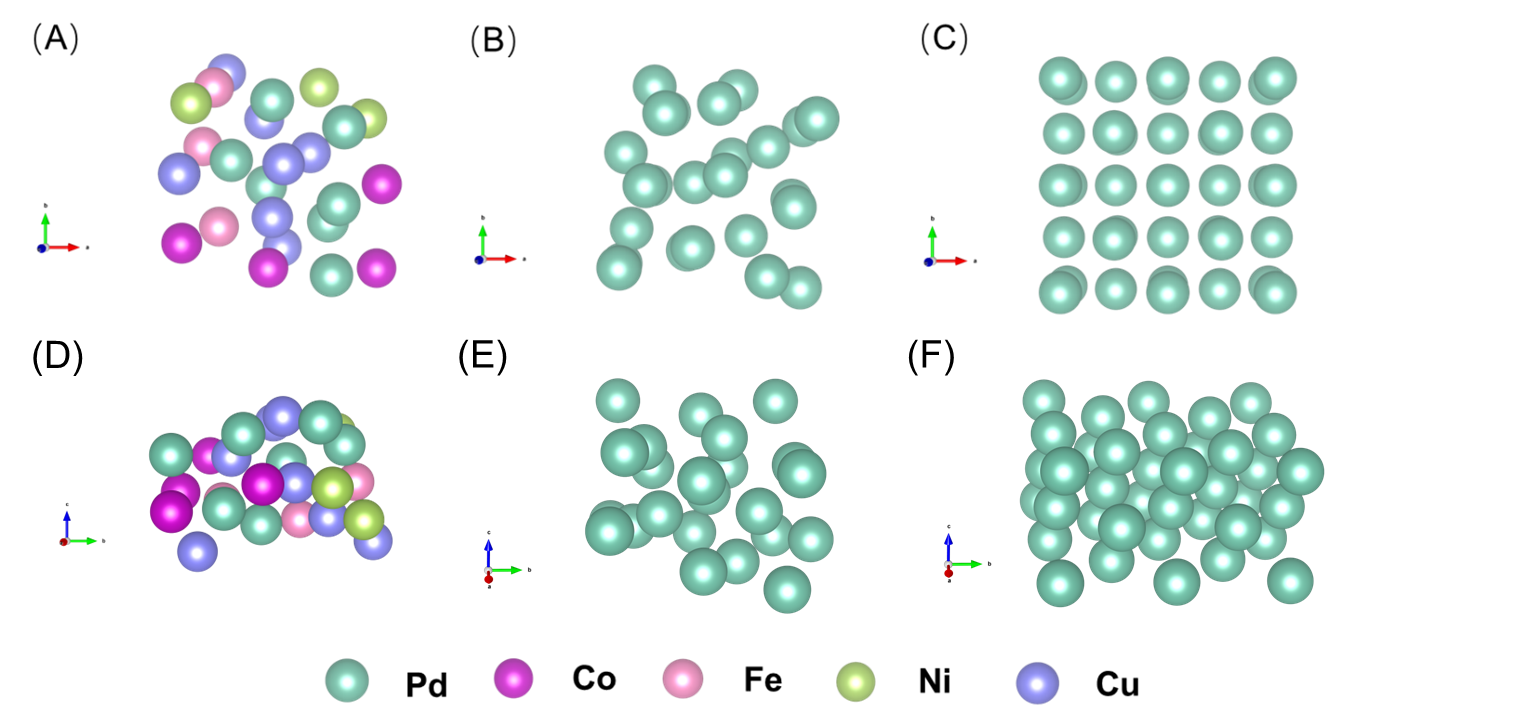


**Figure S33.** Optimized structural models view from top (A-C) and side (D-F) of PdCuNiCoFe HEA (A, D), amorphous Pd (B, E), and crystalline Pd (C, F).


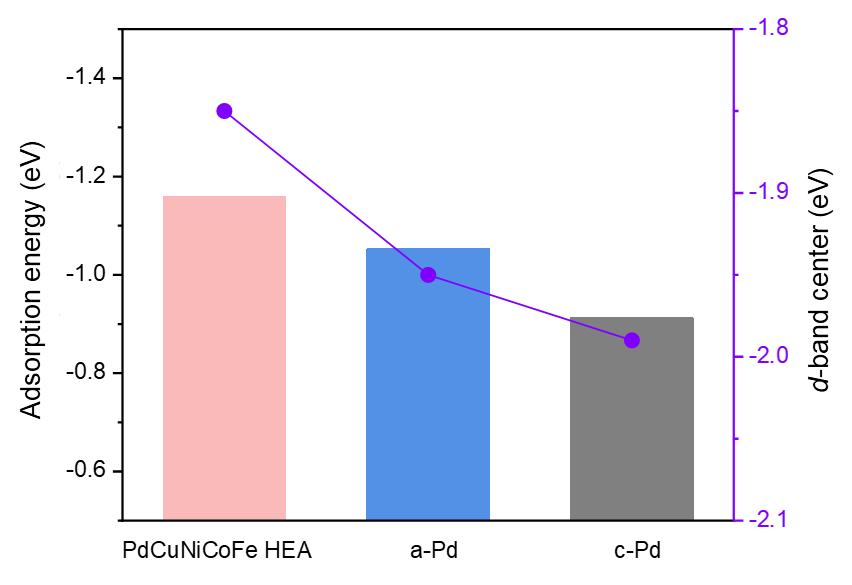


**Figure S34**. The adsorption energy of *EG versus the *d*-band center for the different surfaces.

**
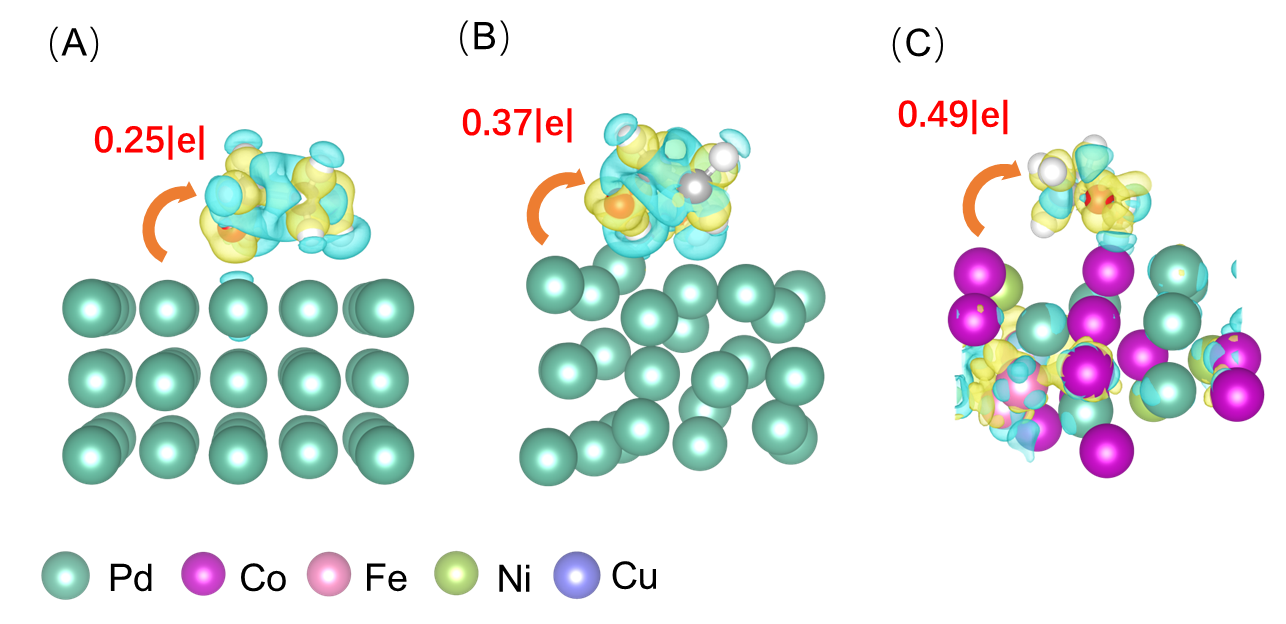
**

**Figure S35.** Charge density differences on the surfaces of (A) crystalline Pd, (B) amorphous Pd, and (C) PdCuNiCoFe HEA with adsorbed *EG.


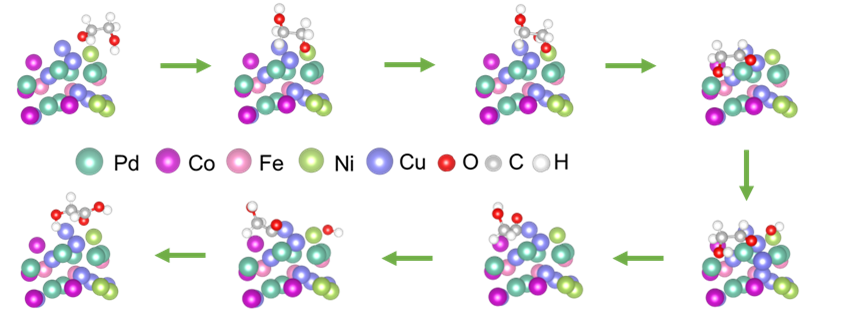


**Figure S36.** Optimized adsorption structures of all intermediates on PdCuNiCoFe HEA during the EGOR pathway.


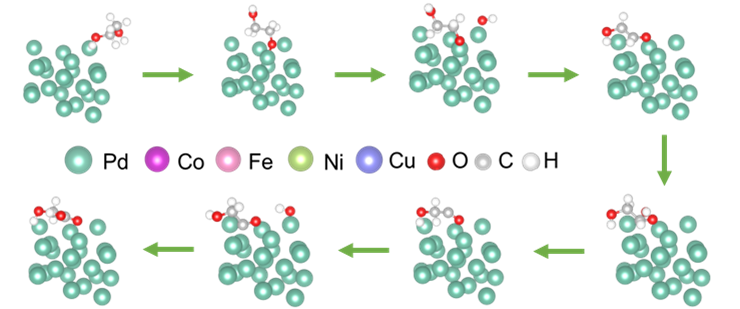


**Figure S37.** Optimized adsorption structures of all intermediates on amorphous Pd during the EGOR pathway.


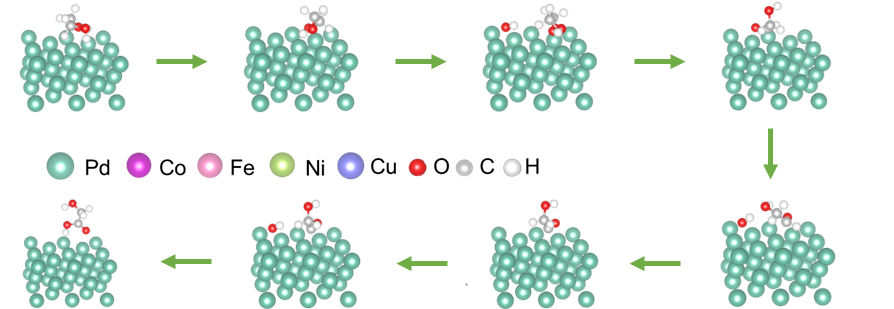


**Figure S38.** Optimized adsorption structures of all intermediates on crystalline Pd during the EGOR pathway.

**Figure S39.** The free energy for C−C bond cleavage during EGOR in different structures.

**Table S1. ICP-OES results.** Atomic ratios of amorphous PdCuNiCoFe HEA nanobranches before and after EGOR, determined by ICP-OES.

|  | **Pd (%)** | **Cu (%)** | **Ni (%)** | **Co (%)** | **Fe (%)** |
| --- | --- | --- | --- | --- | --- |
| **Before EGOR** | 35 | 19 | 22 | 13 | 11 |
| **After EGOR** | 36 | 19 | 22 | 13 | 10 |

**Table S2. EGOR performances of our amorphous PdCuNiCoFe HEA and other reported catalysts.** Comparison of the recently reported noble metal-based electrocatalysts for EGOR under alkaline conditions.

| **Catalysts** | **Electrolyte** | **Activity**  **(A mg_Pd_^−1^)** | **Stability/ Current retention (%)** | **FE for GA (%)** | **Ref.** |
| --- | --- | --- | --- | --- | --- |
| **Amorphous PdCuNiCoFe HEA** | **1.0 M KOH + 1.0 M EG** | **5.8** | **23.9% over 36,000 s** | **98.6** | **This work** |
| PdS nanosheet aerogels | 1.0 M KOH + 0.5 M EG | 2.97 | ~30% over 36,000 s | 83.59 | 9 |
| PdBi alloy | 1.0 M KOH + 0.5 M EG | ~0.6 | / | 99 | 10 |
| Pd-Ni(OH)_2_ | 1.0 M KOH + 1.0 M EG | / | / | 94.1 | 11 |
| Pd-N_4_/Cu-N_4_ | 1.0 M NaOH + xx M EG |  | ~9% over 21,600 s | >80% | 12 |
| Pd-Bi_2_Te_3_ doughnut/Pd hollow nanospheres | 1.0 M KOH + 1.0 M EG | 2.42 | 25.6% over 3,600 s |  | 13 |
| Pt/Ir hetero-metallene | 1.0 M KOH + 1.0 M EG | 0.36 | ~5% over 10,000 s | 87% | 14 |
| Fullerenolamine-modified Pd metallene | 1.0 M KOH + 1.0 M EG | 4.064 | / | / | 15 |
| Lamellar mesoporous PdCu | 1.0 M KOH + 0.1 M EG | 0.422 | / | 96.8 | 16 |
| Au@Pd nanorods | 0.5 M KOH + 0.5 M EG | 5.40 | ~30% over 3,600 s | / | 17 |
| PdNi | 1.0 M KOH + 1.0 M EG | / | / | 96.6 ± 1.7% | 18 |
| Pt–Ni(OH)_2_ | 1.0 M KOH + 0.1 M EG | / | / | 93 | 19 |

**Note:** EG: ethylene glycol; GA: glycolate; FE: Faradaic efficiency;

**Table S3.**  **Structural Details.** The structural parameters of c-Pd and a-Pd models.

|  | Layers | | | Slab thickness (Å) | Vacuum layer thickness (Å) | Fixed layers during relaxation |
| --- | --- | --- | --- | --- | --- | --- |
| c-Pd | | 3 | 3.9 | | 20 | 0 |
| a-Pd | | 3 | 5.17 | | 20 | 0 |

**References**

1. G. Kresse, J. Furthmüller, Efficiency of ab-initio total energy calculations for metals and semiconductors using a plane-wave basis set, *Comput. Mater. Sci.* **6**, 15-50 (1996).
2. P. E. Blöchl, Projector augmented-wave method, *Phys. Rev. B* **50**, 17953 (1994).
3. J. P. Perdew, K. Burke, M. Ernzerhof, Generalized gradient approximation made simple, *Phys. Rev. Lett.* **77**, 3865 (1996).
4. O. Peláez, L. Contreras, M. Balcázar, J. García, L. Arriaga, N. Arjona, Synthesis of a small amorphous PdMo/C nanocatalyst and Pd nanocubes enclosed within (100) planes and their use for ethylene glycol electro–oxidation, *ChemElectroChem* **4**, 728-737 (2017).
5. C. Tang, N. Zhang, Q. Shao, X. Huang and X. Xiao, Rational design of ordered Pd–Pb nanocubes as highly active, selective and durable catalysts for solvent-free benzyl alcohol oxidation, *Nanoscale*, **11**, 5145-5150 (2019).
6. J. Kang, X. Yang, Q. Hu, Z. Cai, L. Liu, L. Guo, Recent Progress of Amorphous Nanomaterials, *Chem. Rev.* **123**, 8859–8941 (2023).
7. J. K. Nørskov, J. Rossmeisl, A. Logadottir, L. Lindqvist, J. R. Kitchin, T. Bligaard, H. Jónsson, Origin of the overpotential for oxygen reduction at a fuel-cell cathode, *J. Phys. Chem. B* **108**, 17886-17892 (2004).
8. Y. Li, Q. Liao, P. Ji, S. Jie, C. Wu, K. Tong, M. Zhu, C. Zhang, H. Li, Accelerated Selective Electrooxidation of Ethylene Glycol and Inhibition of C–C Dissociation Facilitated by Surficial Oxidation on Hollowed PtAg Nanostructures via In Situ Dynamic Evolution, *JACS Au*  **5**, 714-726 (2025).
9. L. Guan, Z. Huang, T. Zhu, Z. Wang, W. Huang, C. Chang, M. Yeh, S. Osella, N. Zhang, T. Liu, Phase-Controlled Pd-S Nanosheet Aerogels for Electrocatalytic Upgrading of Waste Plastics, *Adv. Funct. Mater.* **35**, 2500254 (2025).
10. S. Kang, W. Yuan, X. Guo, Y. Zhang, J. Shang, P. Yang, Y. Ma, V. Nicolosi, L. Cai, B. Qiu, Concurrent Production of Glycolic Acid via Anode Valorization of Plastic Paired With Cathode Upcycling of Biomass Derivative, *Angew. Chem. Int. Ed.* **137**, e202504993 (2025).
11. F. Liu, X. Gao, R. Shi, Z. Guo, E. C. M. Tse, Y. Chen, Concerted and Selective Electrooxidation of Polyethylene-Terephthalate-Derived Alcohol to Glycolic Acid at an Industry-Level Current Density over a Pd-Ni(OH)_2_ Catalyst, *Angew. Chem. Int. Ed.* **62**, e202300094 (2023).
12. E. Moges, C. Chang, W. Huang, K. Lakshmanan, Y. Awoke, C. Pao, M. Tsai, W. Su, B. Hwang, Sustainable Synthesis of Dual Single-Atom Catalyst of Pd-N_4_/Cu-N_4_ for Partial Oxidation of Ethylene Glycol, *Adv. Funct. Mater.* **32**, 2206887 (2022).
13. H. Xu, B. Huang, Y. Zhao, G. He, H. Chen, Engineering Heterostructured Pd–Bi_2_Te_3_ Doughnut/Pd Hollow Nanospheres for Ethylene Glycol Electrooxidation, *Inorg. Chem.* **61** 4533-4540 (2022).
14. K. Deng, Z. Lian, W. Wang, J. Yu, H. Yu, Z. Wang, Y. Xu, L. Wang, H. Wang, Lattice Strain and Charge Redistribution of Pt Cluster/Ir Metallene Heterostructure for Ethylene Glycol to Glycolic Acid Conversion Coupled with Hydrogen Production, *Small* **20**, 2305000 (2024).
15. S. Xie, J. Fu, Q. Huang, W. Yang, A. Yu, Y. Yan, Z. Li, P. Peng, Y. Yin, H. Wang, L. Echegoyen, F. Li, Electronic Modulation and Active Site Exposure Using C60 Fullerenolamine Enable High-Performance Alcohol Oxidation on Pd Metallene Catalysts, *Angew. Chem. Int. Ed.* **137**, e202506044 (2025).
16. S. Han, L. Sun, D. Fan, B. Liu, Pulsed electrosynthesis of glycolic acid through polyethylene terephthalate upcycling over a mesoporous PdCu catalyst, *Nat. Commun.* **16**, 3426 (2025).
17. X. Zhou, Y. Ma, Y. Ge, S. Zhu, Y. Cui, B. Chen, L. Liao, Q. Yun, Z. He, H. Long, L. Li, B. Huang, Q. Luo, L. Zhai, X. Wang, L. Bai, G. Wang, Z. Guan, Y. Chen, C.-S. Lee, J. Wang, C. Ling, M. Shao, Z. Fan, H. Zhang, Preparation of Au@Pd Core–Shell Nanorods with fcc-2H-fcc Heterophase for Highly Efficient Electrocatalytic Alcohol Oxidation, *J. Am. Chem. Soc.*, **144**, 547-555 (2022).
18. S. Zhang, K. Li, X. Zhang, Y. Ye, T. Shi, Y. Jiang, L. Zheng, Y. Lin, H. Zhang, Concurrently Selective Electrosynthesis of Ammonia and Glycolic Acid Over Cathodic Single-Atom Cobalt and Anodic PdNi Alloying Catalysts, *Adv. Funct. Mater.*, **35**, 2415046 (2025).
19. X. Liu, X. He, D. Xiong, G. Wang, Z. Tu, D. Wu, J. Wang, J. Gu, Z. Chen, Electro-Reforming of PET Plastic to C2 Chemicals with Concurrent Generation of Hydrogen and Electric Energy, *ACS Catal.* **14**, 5366–5376 (2024).
